# Defective ALC1 nucleosome remodeling confers PARPi sensitization and synthetic lethality with HRD

**DOI:** 10.1016/j.molcel.2020.12.006

**Published:** 2021-02-18

**Authors:** Graeme Hewitt, Valerie Borel, Sandra Segura-Bayona, Tohru Takaki, Phil Ruis, Roberto Bellelli, Laura C. Lehmann, Lucia Sommerova, Aleksandra Vancevska, Antonia Tomas-Loba, Kang Zhu, Christopher Cooper, Kasper Fugger, Harshil Patel, Robert Goldstone, Deborah Schneider-Luftman, Ellie Herbert, Gordon Stamp, Rachel Brough, Stephen Pettitt, Christopher J. Lord, Stephen C. West, Ivan Ahel, Dragana Ahel, J. Ross Chapman, Sebastian Deindl, Simon J. Boulton

**Affiliations:** 1 The Francis Crick Institute, 1 Midland Road, London NW1 1AT, UK; 2 Department of Cell and Molecular Biology, Science for Life Laboratory, Uppsala University, 75124 Uppsala, Sweden; 3 Medical Research Council (MRC) Molecular Haematology Unit, Weatherall Institute of Molecular Medicine, University of Oxford, Oxford OX3 9DS, UK; 4 Wellcome Centre for Human Genetics, University of Oxford, Oxford OX3 7BN, UK; 5 Centro Nacional de Investigaciones Cardiovasculares (CNIC), Madrid, Spain; 6 Sir William Dunn School of Pathology, South Parks Road, University of Oxford, Oxford OX1 3RE, UK; 7 The CRUK Gene Function Laboratory, The Breast Cancer Now Toby Robins Research Centre, The Institute of Cancer Research, London SW3 6JB, UK; 8 Artios Pharma Ltd., Meditrina, Babraham Research Campus, Cambridge CB22 3AT, UK

**Keywords:** ALC1, chromatin remodeler, DNA damage repair, poly(ADP)-ribosylation, PARPs, base excsion repair, DNA gycosylases, homologous recombination defieciency, BRCAs, synthetic lethality

## Abstract

Chromatin is a barrier to efficient DNA repair, as it hinders access and processing of certain DNA lesions. ALC1/CHD1L is a nucleosome-remodeling enzyme that responds to DNA damage, but its precise function in DNA repair remains unknown. Here we report that loss of ALC1 confers sensitivity to PARP inhibitors, methyl-methanesulfonate, and uracil misincorporation, which reflects the need to remodel nucleosomes following base excision by DNA glycosylases but prior to handover to APEX1. Using CRISPR screens, we establish that ALC1 loss is synthetic lethal with homologous recombination deficiency (HRD), which we attribute to chromosome instability caused by unrepaired DNA gaps at replication forks. In the absence of ALC1 or APEX1, incomplete processing of BER intermediates results in post-replicative DNA gaps and a critical dependence on HR for repair. Hence, targeting ALC1 alone or as a PARP inhibitor sensitizer could be employed to augment existing therapeutic strategies for HRD cancers.

## Highlights

• Loss of ALC1 nucleosome remodeling confers PARPi, MMS, and formyl-dU sensitivity
• ALC1 is required after lesion excision by DNA glycosylases prior to APEX1
• Loss of ALC1 is synthetic lethal with homologous recombination deficiency (HRD)
• Endogenous alkylated base damage is a source of synthetic lethality with HRD

## Graphical Abstract

**Figure:**
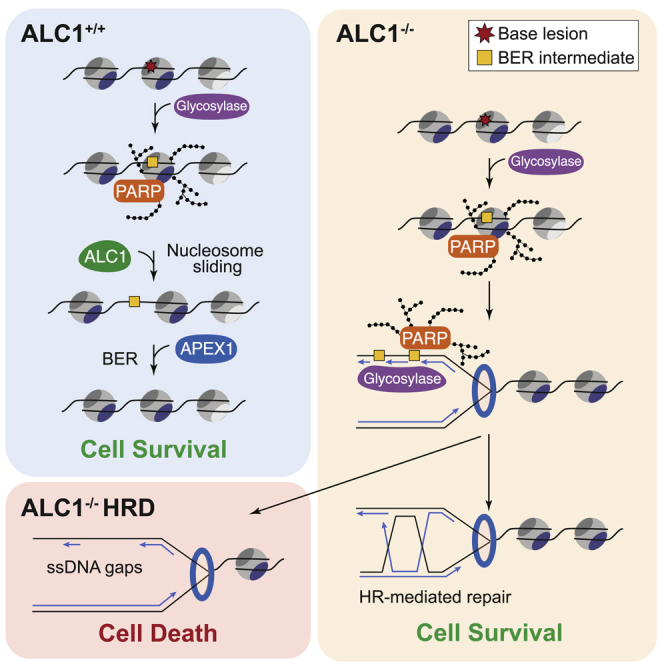

## Teaser

Hewitt et al. report that loss of the chromatin-remodeling enzyme ALC1 leads to persistent BER intermediates and a critical dependency on HR for repair. Combined loss of ALC1 and HR is synthetic lethal, which can be partially rescued by blocking excision of endogenous alkylation damage by the glycosylase MPG.

## Introduction

Repair of DNA double-strand breaks (DSBs) and protection of damaged replication forks is essential for normal cell growth, presents a barrier to cancer development, and shapes the cellular response to radio- and chemotherapies (Chapman et al., 2012). Cancer cells often exhibit altered DNA repair networks, which confer a selective growth advantage to the tumor by potentiating mutator phenotypes and thus driving cancer evolution. Homologous recombination (HR), an essential mechanism of DSB repair and fork protection, is one such DNA repair pathway that is frequently attenuated in cancer. Importantly, deficiencies in the HR pathway create a vulnerability that can be exploited to selectively kill cancer cells by means of synthetic lethality (O’Neil et al., 2017). The paradigm for this approach is the use of poly(ADP)-ribose polymerase inhibitors (PARPi) for the treatment of homologous recombination-deficient (HRD) cancers, which includes breast and ovarian tumors that are mutated in the HR genes *BRCA1* and *BRCA2* (Bryant et al., 2005; Farmer et al., 2005). Despite the notable success of PARPi in the clinic, approximately half of HRD cancers fail to respond to treatment due to innate PARPi resistance, and of those that do respond, >90% ultimately develop acquired PARPi resistance (Noordermeer and van Attikum, 2019). As such, there is an urgent clinical need to identify new therapeutic strategies to improve existing treatments to target HRD and exploit other DNA repair vulnerabilities that exist in cancer, including deficiencies in non-homologous end joining, mismatch repair, base excision repair (BER), and ATM signaling (Gourley et al., 2019).

DNA of eukaryotic cells is compacted into chromatin, and this higher-order complex structure ensures the maintenance of cellular identity. As nucleosomes are perceived as barriers for DNA-related processes, they must first be disassembled or re-organized to allow any DNA-templated machinery to access its substrate. While most bulk chromatin packaging occurs during DNA replication, where histones are evicted ahead of the fork and, together with newly synthesized histones, are re-assembled behind the fork (Hammond et al., 2017), chromatin re-organization outside of S phase impacts on fundamental processes such as transcription and DNA repair. In particular, dynamic changes in chromatin organization occur on damaged chromatin to facilitate timely access of DNA repair enzymes (Price and D’Andrea, 2013). Nucleosome eviction and/or sliding are necessary for chromatin relaxation, as well as prompt nucleosome deposition after removal of the DNA lesion (Ransom et al., 2010). Diverse types of chromatin-remodeling complexes catalyze such chromatin transactions with related ATPase motor translocase domains. Specialized chromatin remodelers involve four subfamilies: imitation switch (ISWI), chromodomain helicase DNA-binding (CHD), switch/sucrose non-fermentable (SWI/SNF), and INO80 (Clapier et al., 2017; Stadler and Richly, 2017). Different subfamilies preferentially achieve particular outcomes, such as facilitating chromatin access for DNA repair transactions, and are targeted to specific chromatin domains via regulatory cues.

ALC1 (amplified in liver cancer 1), also known as CHD1L (chromodomain-helicase-DNA-binding protein 1-like), is an ISWI-related chromatin remodeler encoded by a gene on chromosome 1q21, a region commonly amplified in many cancers (Flaus et al., 2006). ALC1 is differentiated from other members of the ISWI-related remodelers by virtue of a C-terminal macro domain, which possesses high intrinsic affinity for poly(ADP)-ribose (PAR) chains (Ahel et al., 2009). Through its macro domain, ALC1 is rapidly recruited to sites of DNA damage by PAR chains synthesized by PARP1/2 (Ahel et al., 2009; Satoh and Lindahl, 1992). Macro domain binding to PAR chains also relieves an autoinhibitory interaction between the macro and ATPase domains of ALC1, which activates ATP hydrolysis and nucleosome sliding (Lehmann et al., 2017; Singh et al., 2017). ALC1-dependent chromatin remodeling has been proposed to facilitate DNA repair, but evidence in support of this role is currently lacking (Tsuda et al., 2017). Moreover, the precise DNA repair and organismal functions of ALC1 remain unknown.

Here we show that nucleosome remodeling by ALC1 is required downstream of base excision by DNA glycosylases but upstream of APEX1. Loss of ALC1 leads to toxic BER intermediates that result in single-strand gap formation and replication fork collapse. Since blocking this process in cells confers PARPi sensitization and a critical dependence on HR, our study establishes ALC1 as a potential therapeutic target for treating HRD cancers.

## Results

**Figure 1:**
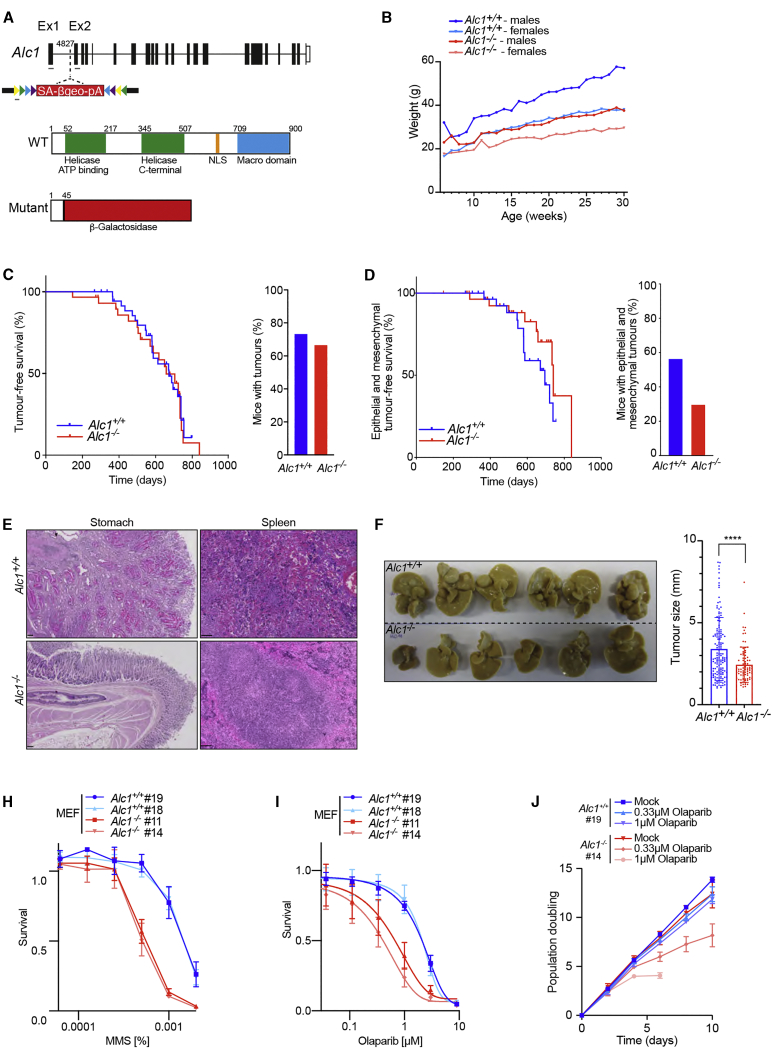
Loss of *Alc1* does not affect lifespan and reduces DEN-induced tumor occurrence (A) Top: Schematic representation of mouse *Alc1* genomic locus. The gene-trap vector rsFROSAbgeo0s is inserted at position 4827 in intron 1. The whole genomic locus is 49.461 kb, and introns (lines) and exons (bars) are approximately to scale. Gray lines represent primers used for genotype. Bottom: ALC1 protein organization. *Alc1* mutant protein is truncated at the 45^th^ amino acid and fused to the β-Geo cassette of the gene-trap vector. (B) Weight analysis of *Alc1*^*+/+*^ and *Alc1*^*−/−*^ mice. Error bars are not shown to render the graph readable; data are from males and females with at least five mice measured at each time point. (C) Tumor-free survival of *Alc1* mice. Significance: Mantel-Cox test, p = 0.4. n = 30 *Alc1*^*+/+*^ and n = 30 *Alc1*^*−/−*^. Mice culled due to nonspecific phenotypes (e.g., dermatitis, overgrown teeth, and fits) were excluded from this study. Right: Frequency of *Alc1* mice that develop tumors. Note that there is no difference between both groups. (D–F) *Alc1*^*−/−*^ mice show reduction in both spontaneous epithelial and mesenchymal and DEN-induced tumor formation. (D) Epithelial and mesenchymal tumor-free survival of *Alc1* mice. Significance: Mantel-Cox test, p = 0.1. n = 30 *Alc1*^*+/+*^ and n = 30 *Alc1*^*−/−*^. Mice culled due to nonspecific phenotypes (e.g., dermatitis, overgrown teeth, and fits) were excluded from this study. Right: Frequency of *Alc1* mice that develop epithelial or mesenchymal tumors. Note that there is a tendency for *Alc1*^*−/−*^ mice to develop less epithelial and mesenchymal tumors. Significance: Fisher’s exact test, p = 0.2. (E) Left: Representative images of epithelial tumors. Note the presence a stomach adenoma with peculiar hyaline pink cells in the *Alc1*^*+/+*^ mouse. Scale bars represent 100 μm. Right: Representative images of mesenchymal tumors. Note the presence an hemangiosarcoma in the spleen of *Alc1*^*+/+*^ mouse. Scale bars represent 100 μm. (F) Left: Pictures of liver from 36-week-old *Alc1*^*+/+*^ and *Alc1*^*−/−*^ male mice intraperitoneally injected with DEN (25 mg/kg body) at 2 weeks of age and fed with high-fat diet. n = 6. Right: Tumor size measurement in mm. Each tumor has been measured with a caliper. Note the smaller-sized tumors in the *Alc1*^*−/−*^ group. Significance: t test, p < 0.0001. (G and H) *Alc1*^−/−^ MEFs are sensitive to PARPi. (G) Reduced survival of *Alc1*^−/−^ MEFs after treatment with Olaparib. Data are mean ± SEM normalized to untreated cells (n = 3 biologically independent experiments). (H) Growth curves in *Alc1*^*+/+*^ and *Alc1*^*−/−*^ MEFs in non-treated controls and with indicated Olaparib doses. Data are mean ± SEM (n = 3 biologically independent experiments). (I) Reduced survival of *Alc1*^−/−^ MEFs after treatment with MMS. Data are mean ± SEM normalized to untreated cells (n = 3 biologically independent experiments).ns, p > 0.05; ^∗^p < 0.05; ^∗∗^p < 0.01; ^∗∗∗^p < 0.001; ^∗∗∗∗^p < 0.0001.

To investigate the role of ALC1 *in vivo*, we derived an *Alc1* knockout mouse model from a gene trap embryonic stem cell line (E305F08) available from the German Gene Trap consortium (GGTC). Mapping by splinkerette PCR located the insertion site within the first intron at position 4827, which is predicted to disrupt the ALC1 protein before the helicase ATP binding domain, leading to a chimeric gene containing part of *Alc1* exon1 fused to β-galactosidase (Figure 1A). This was confirmed by genotyping of wild-type (WT), heterozygous, and mutant mice and the corresponding mouse embryonic fibroblasts (MEFs) (Figures S1A and S1B), and western blotting confirmed loss of ALC1 protein expression (Figure S1C). Although *Alc1*^*−/−*^ mice are viable, they were born at slightly reduced sub-Mendelian ratios (14% versus 25%; Figure S1D) and are of smaller size than their WT littermates throughout adulthood, independent of gender (Figure 1B; Figure S1E). Despite their reduced size, adult *Alc1*^*−/−*^ mice do not present with increased DNA damage in different tissues (Figure S1F) and do not develop any other phenotypic abnormalities that would impact on their total lifespan. Indeed, *Alc1*^*+/+*^ and *Alc1*^*−/−*^ mice have a similar overall survival time (around 600 days; Figure S1G). Hence, loss of ALC1 alone has no effect on lifespan.

To further examine the impact of ALC1 on genome stability and tumorigenesis in mice, we established a tumor watch cohort of 30 *Alc1*^*+/+*^ and 30 *Alc1*^*−/−*^ mice for approximately 22 months. We observed that tumor latency is similar in both groups (673 days for *Alc1*^*+/+*^ versus 658 days for *Alc1*^*−/−*^; Figure 1C). General tumor incidence was also unaffected by loss of ALC1, as 67% of *Alc1*^*−/−*^ mice (20/30) presented with at least one tumor compared to 73% for their WT littermates (22/30). 53% of *Alc1*^*+/+*^ (16/30) versus 43% of *Alc1*^*−/−*^ (13/30) presented with more than one tumor (Figure 1C; Figure S1H). Sub-dividing tumors into three different categories (lymphomas, epithelial, or mesenchymal; Table 1) revealed that although lymphoma-free survival (median survival time: 734 days for *Alc1*^*−/−*^ versus 755 days for *Alc1*^*+/+*^; Figure S1I, left panel) and incidence of lymphomas (Figure S1I, right panel) are similar in *Alc1*^*+/+*^ and *Alc1*^*−/−*^ mice, epithelial or mesenchymal tumor-free survival tended to be increased in *Alc1*^*−/−*^ mice (734 days for mice lacking ALC1 versus 671 days for their WT littermates; Figure 1D, left panel; Figure S1J). Moreover, *Alc1*^*−/−*^ mice exhibited a tendency to develop fewer epithelial or mesenchymal tumors compared to their WT littermates (Figure 1D, right panel). Indeed, 57% of *Alc1*^*+/+*^ mice developed epithelial-mesenchymal tumors whereas only 37% of *Alc1*^*−/−*^ mice were affected. Moreover, epithelial tumor-free survival tended to be slightly increased for mice lacking ALC1, with a median survival of 741 days for *Alc1*^*−/−*^ compared to 695 days for *Alc1*^*+/+*^ (Figure S1J, left panel) accompanied by a mild decrease in the number of *Alc1*^*−/−*^ mice developing one or more than one tumor (Figure S1J, middle and right panel). Interestingly, *Alc1*^*−/−*^ mice do not appear to develop mesenchymal tumors (Figure S1K). These data suggest that lack of ALC1 may protect against development of epithelial and mesenchymal tumors.

To further test this hypothesis, we induced the formation of epithelial tumors in livers of both *Alc1*^*+/+*^ and *Alc1*^*−/−*^ mice via a single intraperitoneal administration of the carcinogen diethylnitrosamine (DEN; 25 mg/kg body weight) into 2-week-old mice followed by feeding with a high-fat diet until the mice reached 36 weeks of age. This study revealed that *Alc1*^*−/−*^ mice developed significantly smaller liver tumors than *Alc1*^*+/+*^ mice (Figure 1F) following DEN injection. Furthermore, histology analysis showed that all tumors are hepatocellular adenomas (Figure S1L), with a moderate decrease in number in *Alc1*-deficient mice suggesting that *Alc1*^*−/−*^ mice are less susceptible to development of epithelial liver tumors than WT animals. Hence, ALC1 loss in mice confers a tendency toward reduced tumor burden.

To determine if and how DNA repair processes are altered in the absence of ALC1, we exposed *Alc1*^*−/−*^ MEFs to genotoxins. While we observed no differences in proliferative capacity between *Alc1*^*+/+*^ and *Alc1*^*−/−*^ MEFs under normal growth conditions, *Alc1*^*−/−*^ MEFs exhibited sensitivity to the alkylating agent methyl-methanesulfonate (MMS) (Figure 1H). Unexpectedly, given that ALC1 is recruited to sites of DNA damage in a PAR-dependent manner, *Alc1*^*−/−*^ MEFs also showed exquisite PARPi sensitivity as measured by cell survival or proliferative capacity (Figures 1I and 1J). Depletion of ALC1 has also scored as sensitizing human cells to PARPi in unbiased CRISPR screens, but the basis of this was unclear (Liu et al., 2020; Zimmermann et al., 2018).

**Figure 2:**
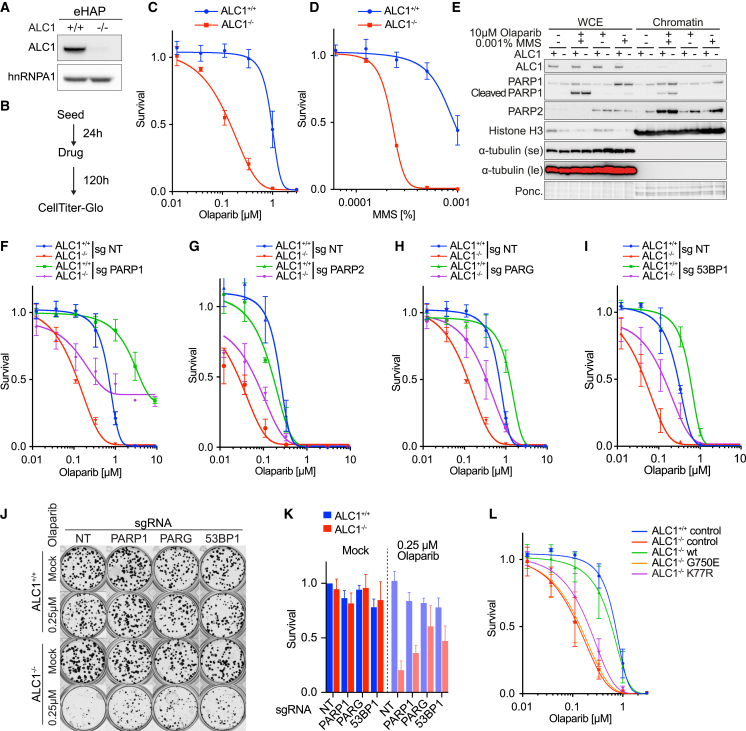
Defective PAR-binding and nucleosome remodeling confers PARPi and MMS sensitivity (A–D) *ALC1*^*−/−*^ cells are sensitive to PARPi and MMS. (A) CRISPR-mediated inactivation of ALC1 in eHAP. Immunoblot of WCEs in *ALC1*^*+/+*^ and *ALC1*^*−/−*^ cells, probed for ALC1. hnRNPA1 was used as a loading control. (B) Schematic representation of survival assays using CellTiter-Glo. (C and D) Reduced survival of eHAP ALC1^−/−^ cells after treatment with indicated genotoxin. Data are mean ± SEM normalized to untreated cells (n = 3 biologically independent experiments). Solid lines show a nonlinear least-squares fit to a four-parameter dose-response model. (E and F) PARP trapping contributes to Olaparib sensitivity in *ALC*^*+/+*^ and *ALC1*^*−/−*^ cells. (E) Immunoblot of WCEs versus chromatin in *ALC1*^*+/+*^ and *ALC1*^*−/−*^ cells following indicated treatments, probed for ALC1, PARP1, and PARP2. α-tubulin was used as a loading control for cytoplasmic fraction. Histone H3 was used as a loading control for chromatin fraction (data are representative of n = 3 biologically independent experiments). (F) Rescue of Olaparib sensitivity in inducible CAS9 (*iCAS9*) *ALC1*^*+/+*^ and *ALC1*^*−/−*^ eHAP-expressing PARP1 sgRNA following 72 h Dox induction. Data are mean ± SEM normalized to untreated cells (n = 3 biologically independent experiments). Solid lines show a nonlinear least-squares fit to a four-parameter dose-response model. (G) Rescue of Olaparib sensitivity in *iCAS9* ALC1^−/−^ eHAP-expressing PARP2 sgRNA following 72 h Dox induction. Data are mean ± SEM normalized to untreated cells (n = 3 biologically independent experiments). Solid lines show a nonlinear least-squares fit to a four-parameter dose-response model. (H) Rescue of Olaparib sensitivity in *iCAS9 ALC1*^*+/+*^ and *ALC1*^*−/−*^ eHAP-expressing PARG sgRNA following 72 h Dox induction. Data are mean ± SEM normalized to untreated cells (n = 3 biologically independent experiments). Solid lines show a nonlinear least-squares fit to a four-parameter dose-response model. (I) Rescue of Olaparib sensitivity in *iCAS9 ALC1*^*+/+*^ and *ALC1*^*−/−*^ eHAP-expressing 53BP1 sgRNA following 72 h Dox. Data are mean ± SEM normalized to untreated cells (n = 3 biologically independent experiments). Solid lines show a nonlinear least-squares fit to a four-parameter dose-response model. (J) Representative images (n = 3 biologically independent experiments) of clonogenic survival assays in *ALC1*^*+/+*^ and *ALC1*^*−/−*^*iCAS9* cells expressing indicated sgRNA following 72 h Dox ± 250 nM Olaparib. (K) Quantification of clonogenic survival assays in ALC1^+/+^ and ALC1^−/−^*iCAS9* cells expressing indicated sgRNAs following 72 h Dox ± 250 nM Olaparib. Data are mean ± SEM normalized to non-treated *ALC1*^*+/+*^ NT sgRNA (n = 3 biologically independent experiments). (L) Olaparib sensitivity is associated with defective nucleosome remodeling. *ALC1*^*+/+*^ and *ALC1*^*−/−*^ eHAP transduced with indicated constructs. Data are mean ± SEM normalized to untreated cells (n = 3 biologically independent experiments). Solid lines show a nonlinear least-squares fit to a four-parameter dose-response model.

To confirm these findings in human cells, we examined the response of diploid *ALC1*^*+/+*^ and *ALC1*^*−/−*^ eHAP cells to a range of DNA-damaging agents (Figures 2A–2D; Figures S2A–S2G). Similar to what we observed in MEFs (Figures 1H–1J), loss of ALC1 conferred exquisite sensitivity to the PARPi Olaparib (Figures 2A–2C), Veliparib, and Talazoparib (Figures S2A and S2B). Further screening of genotoxic compounds confirmed sensitivity to MMS (Figure 2D) and revealed mild sensitivity to hydroxyurea (HU; Figure S2C). However, *ALC1*^*−/−*^ eHAP cells were not sensitive to camptothecin (CPT), aphidicolin, etoposide, or cisplatin (Figures S2D–S2G), indicating that ALC1 is dispensable for the repair of the DNA lesions induced by these genotoxins. Importantly, sensitivity to Olaparib and MMS was also observed in *ALC1*^*−/−*^ U2OS cells (Figures S2H–S2J) and *Alc1*^*−/−*^ MEFs (Figures 1H–1J), indicating that the selective genotoxin sensitivity is not cell-type or species specific.

### Loss of ALC1 confers enhanced PARP trapping on chromatin

PARPi toxicity has been ascribed to both the catalytic inhibition of PARP1 and the trapping of PARPs on chromatin (Bryant et al., 2005; Farmer et al., 2005; Lord and Ashworth, 2017; Murai et al., 2012). To determine how PARP trapping contributes to PARPi toxicity in *ALC1*^−/−^ cells, we conducted stringent chromatin fractionations to assess the levels of PARP1 and PARP2 on chromatin. These experiments revealed increased PARP1 and PARP2 trapping in *ALC1*^−/−^ cells relative to WT controls (Figure 2E), which was further increased upon treatment with Olaparib, MMS, or a combination (Figure 2E). Furthermore, deletion of PARP1 conferred PARPi resistance in both *ALC1*^*+/+*^ and *ALC1*^−/−^ cells (Figure 2F; Figures S2K–S2M), which confirmed that PARP trapping contributes to but is not solely responsible for the ALC1 loss/PARPi synthetic phenotype. Notably, PARP1 depletion increased sensitivity to MMS in both *ALC1*^*+/+*^ and *ALC1*^*−/−*^ cells (Figure S2N), indicating that increased PARP1 trapping (Figure 2E) is a consequence but not the cause of MMS toxicity in these cells. These data suggest that PARP1 trapping is a general mechanism of PARPi toxicity that is independent of ALC1 status.

To investigate the contribution of PARP2 trapping described in Figure 2E to PARPi toxicity, we measured PARPi sensitivity in both *ALC1*^*+/+*^ and *ALC1*^−/−^ cells following knockout of PARP2. Loss of PARP2 alone had no effect on the viability of *ALC1*^*+/+*^ and *ALC1*^−/−^ cells (Figures S2O and S2P). Interestingly, knockout of PARP2 led to increased PARPi sensitivity in *ALC1*^*+/+*^ cells but conversely suppressed PARPi sensitivity in *ALC1*^*−/−*^ cells (Figure 2G). Loss of PARP2 had little effect on MMS sensitivity in *ALC1*^*+/+*^ and *ALC1*^−/−^ cells (Figure S2Q). These data indicate that increased PARP2 trapping contributes to the increased PARPi sensitivity observed in *ALC1*^−/−^ cells.

### ALC1 protects PAR chains from degradation by PARG

A previous study observed that overexpression of ALC1 or the ALC1 macro domain alone (Ahel et al., 2009) results in increased levels of PAR chains in cells, which are normally degraded by the PAR-glycosylase, PARG (Lin et al., 1997; Slade et al., 2011). We therefore considered the possibility that the ALC1 macro domain might bind and protect PAR chains from PARG degradation. Indeed, purified ALC1 macro domain, when incubated with ADP-ribosylated PARP1 or PARylated nucleosomes, protected PAR chains from degradation by human PARG (Figures S2T and S2U). In contrast, PAR protection was attenuated with the PAR-binding mutant ALC1-macro D723A (Figure S2T). Hence, binding by the macro domain of ALC1 protects PAR chains from degradation by PARG *in vitro*. Interestingly, depletion of PARG partially rescued sensitivity to Olaparib in both *ALC1*^*+/+*^ and *ALC1*^*−/−*^ cells (Figure S2R; Figure 2H), suggesting that loss of PARG does not require ALC1 to confer PARPi resistance. Notably, PARG depletion showed a much more pronounced rescue of MMS sensitivity in *ALC1*^*−/−*^ cells when compared to *ALC1*^*+/+*^ (Figure S2S), suggesting that loss of PAR protection by ALC1 may contribute to MMS sensitivity in *ALC1*^*−/−*^ cells.

Since loss of 53BP1 has been shown to permit DSB resection and restore HR in *BRCA1*-deficient tumors, which is one source of PARPi resistance (Bunting et al., 2010), we investigated if alterations in 53BP1 impact the PARPi sensitivity of *ALC1*^*−/−*^ cells. Notably, deletion of 53BP1 (Figure S2V) conferred PARPi resistance in both *ALC1*^*+/+*^ and *ALC1*^*−/−*^ cells but did not affect MMS sensitivity (Figure 2I; Figure S2W). Colony-forming assays (CFAs) confirmed suppression of Olaparib sensitivity following loss of PARP1, PARG, and 53BP1 (Figures 2J and 2K). Furthermore, loss of PARP1 did not confer synthetic lethality with ALC1 deficiency (Figures 2J and 2K).

Finally, complementation of *ALC1*^*−/−*^ cells with WT, but not a PAR-binding mutant ALC1-G750E (Singh et al., 2017) nor a nucleosome-remodeling-deficient ATPase-dead ALC1-K77R mutant (Ahel et al., 2009), was able to rescue sensitivities to both PARPi and MMS (Figure 2L; Figures S2X–S2AB). These data establish that PARPi sensitivity in *ALC1*^*−/−*^ cells reflects a requirement for both PAR binding and nucleosome remodeling and is caused by increased PARP trapping but not loss of PARP enzymatic activity.

Our data indicate that PARPi toxicity is mediated by PARP1 trapping in both *ALC1*^*+/+*^ and *ALC1*^*−/−*^ cells. Similarly, increasing basal levels of PARylation through knockout of PARG or shifting the balance in favor of resection by depletion of 53BP1 rescues PARPi sensitivity independently of ALC1 status. Interestingly, knockout of PARP2 partially rescues PARPi sensitivity specifically in *ALC1*^*−/−*^ but not in *ALC1*^*+/+*^ cells. While these data suggest a role of ALC1 in PARP2 turnover on chromatin, the rescue was incomplete, indicating there are likely additional factors that are responsible for PARPi sensitivity in *ALC1*^*−/−*^ cells. We observe elevated levels of chromatin-bound PARP1 and PARP2 in *ALC1*^*−/−*^ cells even in untreated conditions. We reason that trapping of this increased chromatin-bound population of PARP molecules upon PARPi treatment is responsible for the increased PARPi sensitivity observed in *ALC1*^*−/−*^ cells. However, the mechanism driving the increase in PARP1 and PARP2 on chromatin in *ALC1*^*−/−*^ cells remains unclear.

### Whole-genome CRISPR screens identify ALC1-specific vulnerabilities

ALC1 has previously been suggested to act in the BER pathway, based on epistasis with PARP1 loss and delayed kinetics of single-strand break repair (Tsuda et al., 2017). To test if PARPi sensitivity observed in *ALC1*^*−/−*^ cells could be explained by a loss of BER activity, we generated inducible knockouts of BER genes in *ALC1*^*+/+*^ and *ALC1*^*−/−*^ cell lines (Figure S3A). Analysis of single and double knockout cell lines did not show evidence of growth impairment or synthetic lethality between ALC1 and any of the BER gene knockouts tested (Figures S3B and S3C). Moreover, PolB-, EXO1-, and LIG3-depleted cells showed moderate PARPi sensitivity in CFAs (Figures S3B and S3C), and their loss had an additive effect when depleted in *ALC1*^*−/−*^ cells. In contrast, FEN1 depletion had little effect on PARPi sensitivity in either *ALC1*^*+/+*^ or *ALC1*^*−/−*^ cells (Figures S3D–S3H). The lack of epistasis between ALC1 loss and depletion of these BER genes suggests that the sensitivity to PARPi observed in the absence of ALC1 cannot simply be explained by a general loss of BER activity and that the relation between BER and ALC1 is more complex than previously assumed.

**Figure 3:**
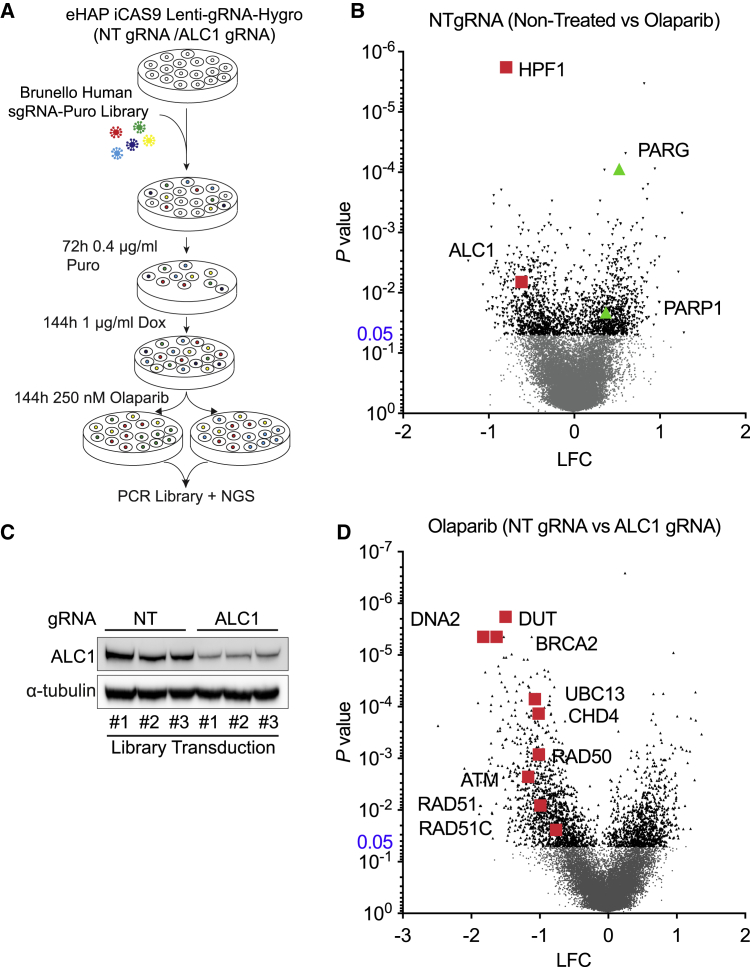
CRISPR screens identify novel synthetic lethalities with ALC1 deficiency (A) Schematic of screening pipeline. (B) Volcano plot of p value versus log-fold change (LFC), iCAS9 eHAP NT gRNA non-treated versus NT gRNA + 250 nM Olaparib. (C) Immunoblot of WCEs in eHAP iCAS9 NT gRNA and ALC1 gRNA from 3 independent biological replicates following 144 h Dox, probed for ALC1; α-tubulin was used as a loading control. (D) Volcano plot of p value versus LFC, iCAS9 eHAP NT gRNA + 250nM Olaparib versus ALC1 gRNA + 250 nM Olaparib.

To investigate the mechanisms of PARPi sensitization and to identify genetic vulnerabilities in *ALC1*^*−/−*^ cells, we performed whole-genome CRISPR screens using inducible Cas9 (iCas9) diploid eHAP cells containing Lenti-gRNA against ALC1 or non-targeting (NT) control, without or treated with 250 nM Olaparib (Figure 3A). Comparison of NT gRNA non-treated versus Olaparib confirmed that ALC1 gRNA sensitizes cells to low-dose Olaparib, while gRNAs targeting PARP1 or PARG conferred PARPi resistance, as documented above (Figure 3B). From the genome-wide screen, histone PARylation factor 1 (HPF1) scored as the top hit when comparing NT gRNA + Olaparib versus NT gRNA + untreated. Loss of HPF1 is known to confer PARPi sensitivity (Gibbs-Seymour et al., 2016) and was recently shown to modulate the catalytic activity of PARP1 (Bilokapic et al., 2020; Suskiewicz et al., 2020). Comparison of NT gRNA + Olaparib versus ALC1 gRNA + Olaparib also revealed that the viability of *ALC1*^*−/−*^ cells is significantly impaired by depletion of DUT, HR factors (BRCA2, RAD51, RAD51C, CHD4), factors that promote DSB resection (RAD50, UBE2N/UBC13, and DNA2), or the DSB-sensing kinase ATM (Figures 3C and 3D).

### ALC1 deficient cells require HR for survival

To validate the hits from the CRISPR screens, we first depleted HPF1 in *ALC1*^*+/+*^ and *ALC1*^*−/−*^ cells to determine if PARPi sensitivity in *ALC1*^*−/−*^ cells is epistatic with HPF1 (Figure S4A). HPF1 loss conferred enhanced sensitivity to PARPi in both *ALC1*^*+/+*^ and *ALC1*^*−/−*^ cells (Figures S4B–S4D) but had little effect on MMS sensitivity (Figure S4E). This suggests that PARPi and MMS sensitivity in *ALC1*^*−/−*^ cells is not mediated by HPF1.

**Figure 4:**
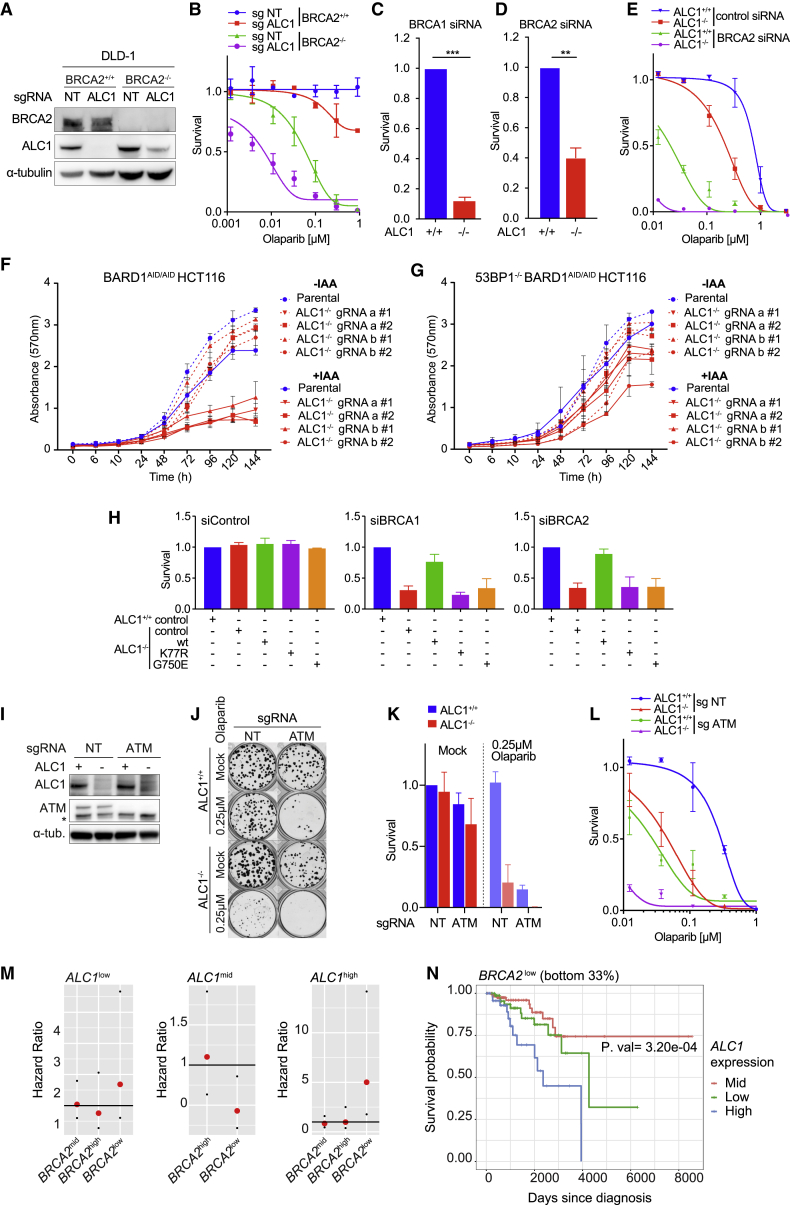
Defective ALC1-mediated nucleosome remodeling confers synthetic lethality with HRD (A–E) Loss of ALC1 is synthetic lethal with HRD and leads to PARPi hypersensitivity. (A) Immunoblot of WCEs from DLD-1 WT and *BRCA2*^*−/−*^ cells following transduction with LentiCRISPR NT sgRNA and ALC1 sgRNA and clonal selection (no BRCA2/ALC1 double knockouts were recovered), probed with BRCA2 and ALC1. α-tubulin was used as a loading control. (B) Olaparib colony survival in DLD-1 *BRCA2*^*+/+*^*ALC1*^*+/+*^, *BRCA2*^*−/−*^*ALC1*^*−/−*^, *BRCA2*^*−/−*^*ALC1*^*+/+*^, and *BRCA2*^*−/−*^*ALC1*^*Low expression*^. Data are mean ± SEM normalized to untreated cells (n = 3 independent biological experiments). Solid lines show a nonlinear least-squares fit to a four-parameter dose-response model. (C) Survival in *ALC1*^*+/+*^ and *ALC1*^*−/−*^ eHAP cells transfected with BRCA1-targeting short interfering RNAs (siRNAs). Cell survival was measured using CellTiter-Glo. Data are mean ± SEM normalized to *ALC1*^*+/+*^ cells (n = 3 independent biological experiments). (D) Survival in *ALC1*^*+/+*^ and *ALC1*^*−/−*^ eHAP cells transfected with BRCA2-targeting siRNAs. Cell survival was measured using CellTiter-Glo. Data are mean ± SEM normalized to *ALC1*^*+/+*^ cells (n = 3 independent biological experiments). (E) Olaparib survival in *ALC1*^*+/+*^ and *ALC1*^*−/−*^ eHAP transfected with non-targeting or BRCA2-targeting siRNAs. Data are mean ± SEM normalized to untreated cells (n = 3 independent biological experiments). Solid lines show a nonlinear least-squares fit to a four-parameter dose-response model. (F) Quantification of a crystal violet proliferation assay in parental and *ALC1-*deleted *BARD1*^*AID/AID*^ cells ± IAA. Data are mean ± SD (n = 3 independent biological experiments). (G) Quantification of a crystal violet proliferation assay in parental and *ALC1-*deleted *53BP1*^*−/−*^*BARD1*^*AID/AID*^ cells ± IAA. Data are mean ± SD (n = 3 independent biological experiments). (H) *ALC1*^+/+^ and *ALC1*^*−/−*^ eHAP cells transduced with indicated ALC1 constructs were transfected with non-targeting, BRCA1-targeting, or BRCA2-targeting siRNAs. Cell survival was measured using CellTiter-Glo. Data are mean ± SEM normalized to *ALC1*^*+/+*^ cells for each siRNA (n = 3 independent biological experiments). (I) Immunoblot of WCEs in *ALC1*^*+/+*^ and *ALC1*^*−/−*^ iCAS9 eHAP cells transduced with ATM sgRNA following 72 h Dox, probed with antibodies against ALC1 and ATM. α-tubulin is used as a loading control. (J) Representative images (n = 3 biologically independent experiments) of clonogenic survival assays in *ALC1*^*+/+*^ and *ALC1*^*−/−*^ iCAS9 cells expressing NT and ATM sgRNA following 72 h Dox ± 250 nM Olaparib. (K) Quantification of clonogenic survival assays in *ALC1*^*+/+*^ and *ALC1*^*−/−*^ iCAS9 cells expressing NT sgRNA and ATM sgRNA following 72 h Dox ± 250 nM Olaparib. Data are mean ± SEM normalized to non-treated *ALC1*^*+/+*^ NT sgRNA (n = 3 biologically independent experiments). (L) Olaparib survival of *ALC1*^*+/+*^ and *ALC1*^*−/−*^ iCAS9 cells transduced with NT sgRNA and ATM sgRNA following 72 h Dox. Data are mean ± SEM normalized to untreated cells (n = 3 independent biological experiments). Solid lines show a nonlinear least-squares fit to a four-parameter dose-response model. (M) Hazard ratio analysis of breast cancer patients from TGCA according to ALC1 and BRCA2 expression. (N) KM survival analysis of BRCA2^low^ breast cancer patients from TGCA according to ALC1 expression. ns, p > 0.05; ^∗^p < 0.05; ^∗∗^p < 0.01; ^∗∗∗^p < 0.001; ^∗∗∗∗^p < 0.0001.

Next, we sought to confirm the loss of viability observed with combined depletion of ALC1 and HR factors. To this end, we successfully generated DLD-1 *BRCA2*^*+/+*^ *ALC1*^*−/−*^ cells, but failed to recover any DLD-1 *BRCA2*^*−/−*^ *ALC1*^*−/−*^ clones, reinforcing the notion that ALC1 loss is synthetic lethal with HRD. In further support of this possibility, DLD *BRCA2*^*−/−*^ clones with reduced ALC1 expression (Figure 4A) exhibited severely impaired proliferative capacity and hyper-sensitivity to Olaparib (Figure 4B; Figure S4F). Synthetic lethality between ALC1 and HRD was further suggested in *ALC1*^*−/−*^ cells subject to siRNA against either BRCA1 or BRCA2 (Figures 4C and 4D; Figure S4G). BRCA2 silencing in *ALC1*^*−/−*^ cells also conferred exquisite PARP inhibitor and MMS sensitivity (Figure 4E; Figures S4H–S4J).

To examine if deleting 53BP1 could restore viability in *ALC1*^*−/−*^ BRCA1/BARD1-depleted cells, we generated ALC1 knockouts in WT and *53BP1*-deleted clones of *BARD1*^*AID/AID*^ HCT-116 cells (Figure S4K), a cell line engineered to encode biallelic auxin-dependent degron tags at the BARD1 C terminus (Nakamura et al., 2019). Treatment of cells with inodole-3-acetic acid (IAA) led to rapid and stable BARD1 degradation (Nakamura et al., 2019) (Figure S4G), which resulted in synthetic lethality in *ALC1*^*−/−*^ cells but not in the WT controls (Figure 4F). Notably, knockout of 53BP1 rescued synthetic lethality in *ALC1*^*−/−*^ BARD1-depleted cells (Figure 4G), indicating that restoration of HR is sufficient to rescue synthetic lethality between ALC1 and BRCA1/BARD1 deficiencies. Furthermore, synthetic lethality between ALC1 loss and HRD could be rescued by complementation with WT ALC1, but not by ALC1-G750E or ALC1-K77R mutants (Figure 4H), indicating that both PAR binding and nucleosome-remodeling activities of ALC1 are important in this context.

Our CRISPR dropout screen also identified UBC13 as sensitizing ALC1-deficient cells to PARPi (Figure 3D). Similar to loss of BRCA1, BARD1, or BRCA2, depletion of UBC13, which compromises DSB resection and template switching, reduced the viability of *ALC1*^*−/−*^ cells and conferred hyper-sensitivity to PARPi and MMS (Figures S4M–S4Q). In addition, the DSB activated checkpoint kinase ATM also scored as a hit for sensitizing ALC1-depleted cells to PARPi (Figure 3D). Depletion of ATM conferred sensitivity to PARPi in *ALC1*^*+/+*^cells, with *ALC1*^*−/−*^ cells showing hyper-sensitivity (Figures 4I–4L) and similar sensitivity in response to MMS (Figures S4R). Collectively, these data show that deficiencies in HR, DSB processing, or the DSB-sensing kinase ATM confer synthetic growth defects and PARPi hyper-sensitization when combined with loss of ALC1.

To examine the clinical relevance of ALC1 status in the context of HRD cancers, we next examined if expression levels of ALC1, BRCA1/2, or ATM influence breast cancer survival in human patients. We observed no relationship between ALC1 expression and BRCA1 or ATM in this analysis. Interestingly, we observed a significantly increased hazard ratio in patients with tumors expressing low levels of BRCA2 and high levels of ALC1 (Figure 4M). Moreover, Kaplan-Meier survival analysis revealed poor survival in this cohort (Figure 4N). These data suggest that high levels of ALC1 in BRCA2^low^ tumors results in a more aggressive disease with poorer prognosis. This raises the possibility that targeting ALC1 could improve the survival outcome in patients with HRD cancers.

### Mechanism of ALC1/HRD synthetic lethality

**Figure 5:**
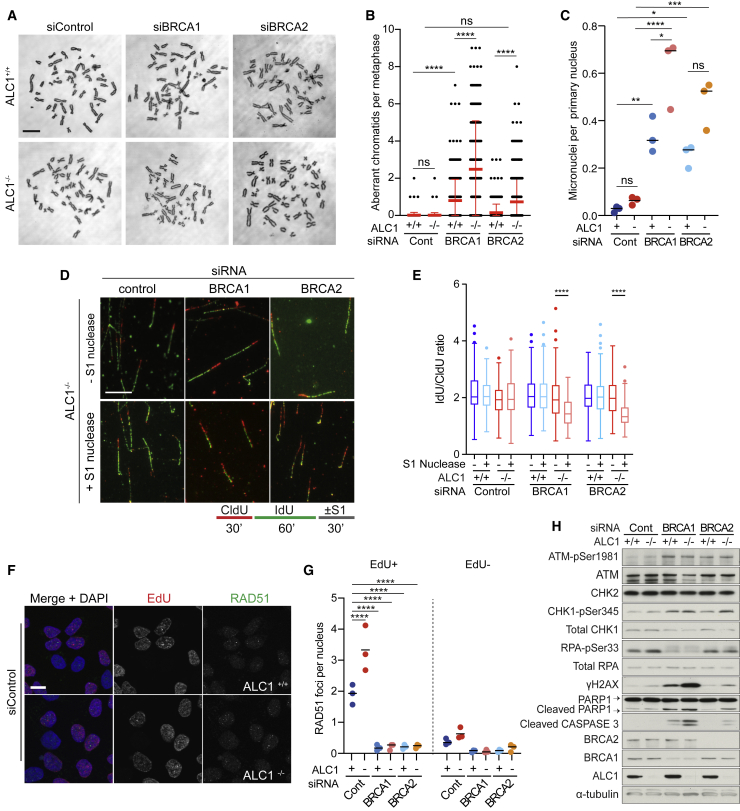
Loss of HR leads to single-stranded gaps at replication forks and gross genomic instability in ALC1^−/−^ cells (A–D) Knockdown of BRCA1/2 in *ALC1*^*−/−*^ cells results in genome instability. (A) Representative micrographs of metaphase spreads in *ALC1*^*+/+*^ and *ALC1*^*−/−*^ eHAP transfected with the indicated siRNA. (B) Quantification of the number of aberrant chromatids per metaphase in *ALC1*^*+/+*^ and *ALC1*^*−/−*^ eHAP transfected with indicated siRNA. Data are mean ± SEM (n = 3 independent biological experiments). (C) Quantification of number of micronuclei per primary nucleus from *ALC1*^*+/+*^ and *ALC1*^*−/−*^ eHAP cells transfected with non-targeting, BRCA1-targeting, and BRCA2-targeting siRNAs. Data are means from individual experiments; bar represents median (n = 3 independent biological experiments). (D) Lower: scheme of the nucleotide labeling and S1 nuclease treatment strategy used for gap detection at the replication fork. Upper: Representative DNA fiber immunofluorescence images from *ALC1*^*−/−*^ eHAP cells transfected with the indicated siRNAs and treated or not with S1 nuclease.Scale bars represent 100 μm. (E) Boxplot showing mean IdU/CldU ratio in *ALC1*^*+/+*^ and *ALC1*^−/−^ eHAP transfected with the indicated siRNAs and treated or not with S1 nuclease. Data from 500–600 fibers/condition are represented as mean ± SD (2 technical replicates from 2 independent biological experiments). (F) Representative micrographs of *ALC1*^*+/+*^ and *ALC1*^*−/−*^ eHAP cells transfected with non-targeting siRNAs stained with RAD51 antibody, EdU click-iT, and DAPI. Scale bar, 10 μm. (G) Quantification of nuclear RAD51 foci in CSK pre-extracted EdU+ and EdU− *ALC1*^*+/+*^ and *ALC1*^*−/−*^ eHAP cells transfected with indicated siRNAs 72 h following knockdown. Data are means from individual experiments; bar represents median (n = 3 biologically independent experiments). (H) Immunoblot of WCEs in *ALC1*^*+/+*^ and *ALC1*^*−/−*^ cells transfected with non-targeting, BRCA1-targeting, or BRCA2-targeting siRNAs, probed with ATM pSer1981, ATM, CHK2, CHK1-pSer345, total CHK1, RPA-pSer33, total-RPA, γH2AX, PARP1, cleaved caspase-3, BRCA2, BRCA1, and ALC1. α-tubulin was used as a loading control. ns, p > 0.05; ^∗^p < 0.05; ^∗∗^p < 0.01; ^∗∗∗^p < 0.001; ^∗∗∗∗^p < 0.0001.

To further explore the basis of synthetic lethality between ALC1 loss and HRD, we first examined metaphase spreads for changes in chromosome integrity. Depletion of BRCA1 or BRCA2 alone conferred the expected increase in chromosome abnormalities on metaphase spreads, consistent with loss of HR. Strikingly, knockdown of BRCA1 or BRCA2 in *ALC1*^*−/−*^ cells resulted in a significant increase in chromosomal abnormalities at metaphase and increased micronuclei when compared to *ALC1*^*+/+*^ cells depleted for HR (Figures 5A–5C; Figure S5A). Cells lacking both ALC1 and HR also presented with a lower percentage of cells in S phase and an increase in the sub-G1 population (Figures S5B and S5C).

Since HR plays a critical role in the protection of damaged replication forks (Ray Chaudhuri et al., 2016; Schlacher et al., 2011), we analyzed replication dynamics in *ALC1*^*+/+*^ and *ALC1*^*−/−*^ cells ± HRD by DNA fiber assay. When compared to control cells, we detected a modest but significant increase in fork speed in *ALC1*^*−/−*^ cells, which has also been reported upon silencing of PARP1 (Maya-Mendoza et al., 2018). Knockdown of BRCA1 or BRCA2 further increased fork speeds in *ALC1*^*−/−*^ cells, suggestive of unrestrained fork progression (Figures S5D and S5E). *ALC1*^*−/−*^ cells depleted for BRCA1 or BRCA2 also presented with a significant increase in replication fork asymmetry relative to controls, which is indicative of increased replication fork stalling and/or collapse (Figures S5E and S5F).

We next considered the possibility that loss of ALC1 might lead to the accumulation of ssDNA gaps during DNA replication, which would place a critical dependence on HR and BRCA1/2 for fork stabilization and repair. To test this hypothesis, we tested for the presence of ssDNA gaps at replication forks using a modified DNA fiber assay, which includes S1 nuclease degradation of single-stranded DNA (ssDNA) (Quinet et al., 2017). Neither *ALC1*^*−/−*^ nor HR-deficient cells exhibited a detectable reduction in the IdU/CldU ratio upon S1 nuclease treatment (Figures 5D and 5E), indicating that ssDNA gaps are not detectable in these contexts. However, since HR is proficient in *ALC1*^*−/−*^ cells, ssDNA gaps could be missed due to repair by HR in this context. Consistent with this possibility, *ALC1*^*−/−*^ cells subjected to siRNA depletion of BRCA1 or BRCA2 exhibited a significant reduction in the IdU/CldU ratio (Figures 5D and 5E), which suggests that ALC1 loss leads to ssDNA gaps that accumulate when HR repair is compromised. In agreement with this conclusion, EdU-positive *ALC1*^*−/−*^ cells showed elevated levels of RAD51 foci, consistent with replication-associated activation of HR (Figures 5F and 5G). Furthermore, knockdown of BRCA1 or BRCA2, which abolish RAD51 focus formation, conferred significant elevation in the number of ssDNA binding protein RPA foci (Figures S5G and S5H), which likely correspond to sites of ssDNA gaps. Accumulation of ssDNA gaps also resulted in activation of a robust DDR, including increased pS1981 ATM, γH2AX and 53BP1 foci, and activation of apoptosis as evident from cleaved caspase-3 and PARP1 cleavage (Figure 5H; Figures S5I–S5N).

### ALC1 is required for the removal of dUTP misincorporation in DNA

**Figure 6:**
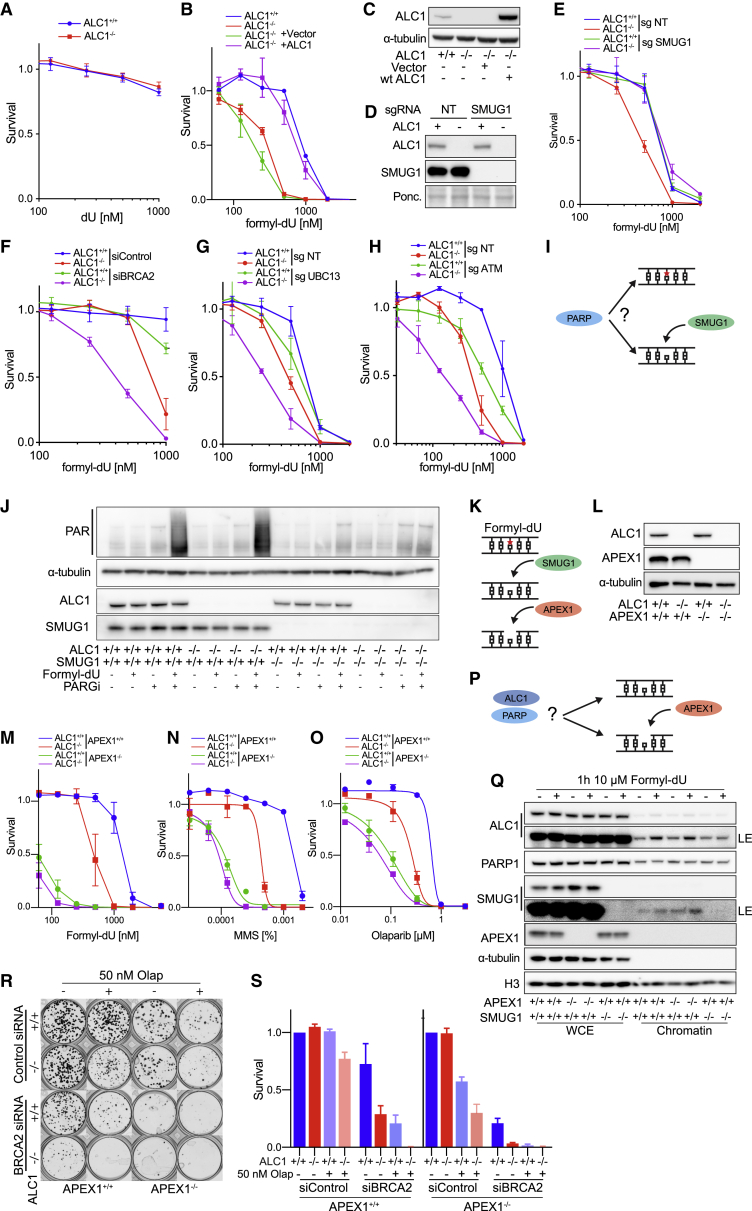
The monofunctional uracil glycosylase SMUG1 and APEX1 mediate formyl-dU sensitivity in ALC1-deficient cells (A) *ALC1*^*+/+*^ and *ALC1*^*−/−*^ eHAP are not sensitive to dU. Data are mean ± SEM normalized to untreated cells (n = 3 independent biological experiments). (B) *ALC1*^*−/−*^ cells are sensitive to formyl-dU. *ALC1*^*+/+*^ and *ALC1*^*−/−*^ eHAP transduced with indicated constructs. Data are mean ± SEM normalized to untreated cells (n = 3 biologically independent experiments). (C) Immunoblot of WCEs in ALC1^*+/+*^ and ALC1^*−/−*^ cells transduced with indicated constructs, probed for ALC1. α-tubulin was used as a loading control. (D and E) SMUG1 knockout rescues ALC1-dependent formyl-dU sensitivity. (D) Immunoblot of WCEs in *ALC1*^*+/+*^ and *ALC1*^*−/−*^*iCAS9* cells transduced with NT sgRNA and SMUG1 sgRNA following 72 h Dox, probed with ALC1 and SMUG1. Ponceau was used as a loading control. (E) Formyl-dU survival of *ALC1*^*+/+*^ and *ALC1*^*−/−*^*iCAS9* cells transduced with NT sgRNA and SMUG1 sgRNA following 72 h Dox. Data are mean ± SEM normalized to untreated cells (n = 3 independent biological experiments). (F) Formyl-dU survival of *ALC1*^*+/+*^ and *ALC1*^*−/−*^ eHAP cells transfected with non-targeting or BRCA2-targeting siRNAs. Data are mean ± SEM normalized to untreated cells (n = 3 biologically independent experiments). (G) Formyl-dU survival of *ALC1*^*+/+*^ and *ALC1*^*−/−*^ eHAP cells expressing NT or UBC13 sgRNA following 72 h Dox. Data are mean ± SEM normalized to untreated cells (n = 3 biologically independent experiments). (H) Formyl-dU survival of *ALC1*^*+/+*^ and *ALC1*^*−/−*^ eHAP cells expressing NT or ATM sgRNA following 72 h Dox. Data are mean ± SEM normalized to untreated cells (n = 3 biologically independent experiments). (I) Is PARylation upstream of formyl-dU (red star) removal by SMUG1? (J) PARylation by PARPs occurs downstream of SMUG1. Immunoblot of WCEs in *ALC1*^*+/+*^*SMUG1*^*+/+*^, *ALC1*^*+/+*^*SMUG1*^*−/−*^, *ALC1*^*−/−*^*SMUG1*^*+/+*^, and *ALC1*^*−/−*^*SMUG1*^*−/−*^ cells with indicated treatments, probed for ALC1, SMUG1, and anti-PAR binding reagent. α-tubulin was used as a loading control. (K) Schematic illustrating (BER) repair of formyl-dU (red star). The monofunctional glycosylase SMUG1 catalyzes the removal of formyl-dU, creating an abasic (AP) site. The endonuclease APEX1 catalyzes the incision of the DNA backbone, leaving a 5′-deoxyribose phosphate. (L) Immunoblot of WCEs in cells with indicated genotypes probed for ALC1 and APEX1. α-tubulin was used as a loading control. (M) Formyl-dU survival of *ALC1*^*+/+*^*APEX1*^*+/+*^, *ALC1*^*+/+*^*APEX1*^*−/−*^, *ALC1*^*−/−*^*APEX1*^*+/+*^, and *ALC1*^*−/−*^*APEX*^*−/−*^ cells. Data are mean ± SEM normalized to untreated cells (n = 3 independent biological experiments). (N) MMS survival of *ALC1*^*+/+*^*APEX1*^*+/+*^, *ALC1*^*+/+*^*APEX1*^*−/−*^, *ALC1*^*−/−*^*APEX1*^*+/+*^, and *ALC1*^*−/−*^*APEX1*^*−/−*^ cells. Data are mean ± SEM normalized to untreated cells (n = 3 independent biological experiments). (O) Olaparib survival of *ALC1*^*+/+*^*APEX1*^*+/+*^, *ALC1*^*+/+*^*APEX1*^*−/−*^, *ALC1*^*−/−*^*APEX1*^*+/+*^, and *ALC1*^*−/−*^*APEX1*^*−/−*^ cells. Data are mean ± SEM normalized to untreated cells (n = 3 independent biological experiments). Solid lines show a nonlinear least-squares fit to a four-parameter dose-response model. (P) Is incision by APEX1 required for ALC1 recruitment? (Q) ALC1 recruitment is upstream of incision by APEX1. Immunoblot of CSK chromatin fractionation in cells with indicated genotype ± formyl-dU treatment, probed for ALC1, PARP1, SMUG1, and APEX1. α-tubulin and histone H3 were used as loading controls. (R) Representative images (n = 3 biologically independent experiments) of clonogenic survival assays in *ALC1*^*+/+*^*APEX1*^*+/+*^, *ALC1*^*+/+*^*APEX1*^*−/−*^, *ALC1*^*−/−*^*APEX1*^*+/+*^, and *ALC1*^*−/−*^*APEX1*^*−/−*^ eHAP cells transfected with non-targeting or BRCA2-targeting siRNAs ± 50 nM Olaparib. (S) Quantification of clonogenic survival assays in *ALC1*^*+/+*^*APEX1*^*+/+*^, *ALC1*^*+/+*^*APEX1*^*−/−*^, *ALC1*^*−/−*^*APEX1*^*+/+*^, and *ALC1*^*−/−*^*APEX1*^*−/−*^ eHAP cells transfected with non-targeting or BRCA2-targeting siRNAs ± 50 nM Olaparib. Data are mean ± SEM normalized to non-treated *ALC1*^*+/+*^*APEX1*^*+/+*^ (n = 3 biologically independent experiments).

Prompted by the results of our CRISPR screen, we next examined the genetic interaction between ALC1 and DUT, an essential enzyme that dephosphorylates dUTP to dUMP and hence provides dUMP as a precursor for thymidine synthesis as well as limiting the intracellular pool of dUTP (Hirmondo et al., 2017). CFAs established that deletion of DUT is lethal in both *ALC1*^*+/+*^ and *ALC1*^*−/−*^ cells (Figures S6A and S6B). Our results from the CRISPR screen showed that, despite being essential, DUT drops out of the ALC1 sgRNA arm faster than in non-targeting sgRNA, suggestive of a negative effect of DUT depletion in *ALC1*^*−/−*^ cells (Figure 3D). Since elevated levels of dUTP lead to increased uracil misincorporation into DNA (Vértessy and Toth, 2009), we considered the possibility that the impact on viability of ALC1^−/−^ cells following depletion of DUT may reflect a defect in dealing with misincorporated uracil in DNA. To test this possibility, *ALC1*^*+/+*^ and *ALC1*^*−/−*^ cells were exposed to the uracil analogs dU, formyl-dU, or FU (Figures 6A and 6B; Figure S6C). This experiment revealed sensitivity of *ALC1*^*−/−*^ cells specifically to formyl-dU (Figure 6B), which results in a misincorporated lesion that is normal excised from DNA by the uracil DNA glycosylase SMUG1 (Masaoka et al., 2003). Re-introduction of WT ALC1 in *ALC1*^*−/−*^ cells restored formyl-dU sensitivity to the same levels as in *ALC1*^*+/+*^ cells, confirming that sensitivity is due to loss of ALC1 (Figures 6B and 6C).

To further investigate a role for ALC1 in uracil removal from DNA *in vivo*, we generated *ALC1*^*+/+*^ and *ALC1*^*−/−*^ cells with inducible knockouts for the uracil glycosylases UNG, MBD4, and SMUG1 (Hashimoto et al., 2012; Haushalter et al., 1999; Krokan et al., 2001) (Figure 6D; Figures S6D and S6E). Surprisingly, depletion of SMUG1 in *ALC1*^*−/−*^ cells rescued formyl-dU sensitivity (Figure 6E), but not sensitivity to MMS or PARPi (Figures S6F and S6G). In contrast, knockout of UNG or MBD4 had no effect on sensitivity to Olaparib, MMS, or formyl-dU (Figures S6H–S6M). UNG knockout did, however, reduce the fitness of *ALC1*^*−/−*^ cells (Figures S6N and S6O). Since SMUG1 can compensate for uracil incision by UNG (Kavli et al., 2002; Nilsen et al., 2001), this toxicity could be driven by increased SMUG1 activity. This suggests that SMUG1 itself creates toxic lesions in the absence of ALC1, at least with respect to formyl-dU. These results also exclude endogenous uracil misincorporation as the source of MMS or PARPi sensitivity in *ALC1*^*−/−*^ cells.

Similar to that observed with MMS (Figure S2N), loss of PARP1 further sensitized both *ALC1*^*+/+*^ and *ALC1*^*−/−*^ cells to formyl-dU (Figure S6P). Knockout of both PARG and 53BP1 partially rescued formyl-dU sensitivity in *ALC1*^*−/−*^ cells only (Figures S6Q and S6R). Interestingly, knockout of PARP2 led to increased formyl-dU sensitivity in *ALC1*^*+/+*^ cells while conferring moderate resistance in *ALC1*^*−/−*^ cells (Figure S6S), similar to that observed with PARPi (Figure 2G). Having observed that sensitivity to PARPi and MMS in ALC1-deficient cells is greatly exacerbated by loss of BRCA1/2, UBC13, or ATM, we also examined if the same is true for sensitivity to formyl-dU. Indeed, loss of BRCA2, UBC13, or ATM led to formyl-dU hypersensitivity in ALC1-deficient cells, which provides further evidence that HR is required as a backup repair pathway in *ALC1*^*−/−*^ cells in response to a range of genotoxin lesions (Figures 6F–6H).

### Loss of ALC1 is epistatic with APEX1

We next sought to understand at which point in the BER pathway ALC1 acts using formyl-dU as a source of DNA lesions. Removal of formyl-dU is catalyzed by SMUG1, creating an abasic (AP) site. Once the AP site is formed, the endonuclease APEX1 catalyzes the incision of the DNA backbone, leaving a 5′-deoxyribose phosphate (Figure 6K).

Since recruitment of ALC1 to damage sites requires PARP-mediated PARylation, we asked whether PARylation occurs as a direct result of formyl-dU misincorporation or is induced following base excision by SMUG1. To this end, we generated SMUG1 knockouts in *ALC1*^*+/+*^ and *ALC1*^*−/−*^ cells and assayed PARylation following treatment with formyl-dU. We also subjected cells to PARGi treatment to block the degradation of any resulting PARylation signal (Figures 6I and 6J). This experiment revealed that PARylation induced in response to formyl-dU requires SMUG1.

To examine the relationship between APEX1 and ALC1, we generated APEX1 knockouts in *ALC1*^*+/+*^ and *ALC1*^*−/−*^ cells. Intriguingly, loss of both ALC1 and APEX1 did not lead to increased sensitivity to formyl-dU, MMS, and PARPi (Figures 6M–6O), indicating epistasis between ALC1 and APEX1. To determine if ALC1 acts upstream or downstream of APEX1, we examined ALC1 recruitment to chromatin following formyl-dU treatment in *APEX*^*+/+*^ and *APEX*^*−/−*^ cells. Treatment of cells with formyl-dU resulted in robust ALC1 recruitment in both *APEX*^*+/+*^ and *APEX*^*−/−*^ cells, indicating that ALC1 acts upstream of or in parallel to APEX1. In contrast, knockout of SMUG1 prevented ALC1 recruitment to chromatin in response to formyl-dU (Figures 6P and 6Q).

Finally, *APEX1*^*−/−*^ and *ALC1*^*−/−*^ cells both showed synthetic lethality with loss of BRCA1/2, with a very modest additive effect in *APEX1*^*−/−*^ *ALC1*^*−/−*^ double knockouts (Figures 6R and 6S). These data suggest that AP site generation by SMUG1 triggers PARylation and ALC1 recruitment, with APEX1 acting in parallel or downstream. Interestingly, a previous report has shown that APEX1 activity is inhibited in the context of the nucleosome *in vitro* (Eccles et al., 2015; Hinz, 2014; Hinz et al., 2015). Our data raise the possibility that nucleosome remodeling by ALC1 may facilitate efficient APEX1 activity *in vivo*.

### MPG causes MMS, PARPi sensitivity, and synthetic lethality with HRD in *ALC1*^*−/−*^ cells

**Figure 7:**
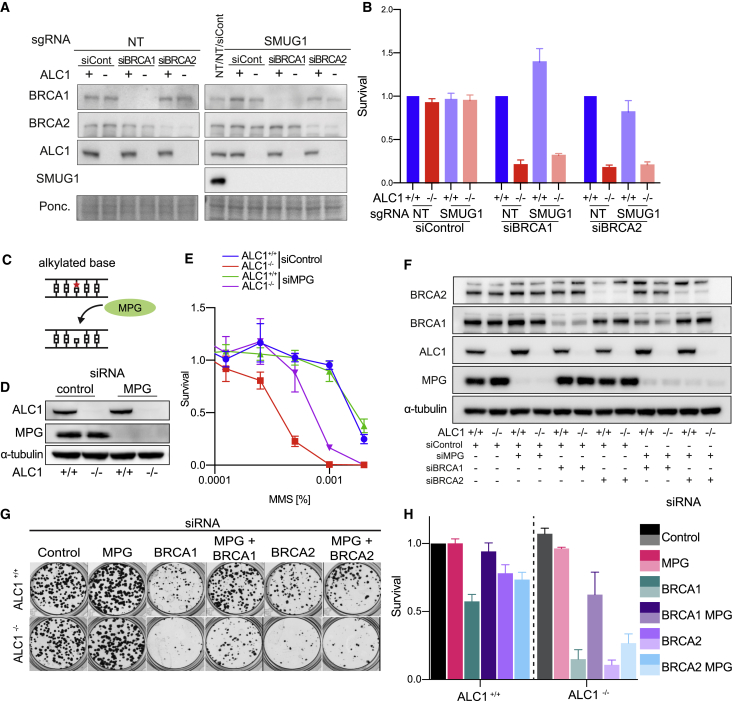
The monofunctional glycosylase MPG drives MMS sensitivity and contributes to synthetic lethality with BRCA1/2 in ALC1-deficient cells (A and B) Processing of endogenous lesions by SMUG1 does not drive synthetic lethality with BRCA1/2 in ALC1-deficient cells. (A) Immunoblot of WCEs in *ALC1*^*+/+*^ and *ALC1*^*−/−*^*i*CAS9 cells transduced with NT sgRNA or SMUG1 sgRNA following 72 h Dox and transfected with non-targeting or BRCA1/2-targeting siRNAs, probed with ALC1, BRCA1, BRCA2, and SMUG1. Ponceau was used as a loading control. (B) Survival in *ALC1*^*+/+*^ and *ALC1*^*−/−*^*iCAS9* cells transduced with NT sgRNA or SMUG1 sgRNA following 72 h Dox and transfected with non-targeting or BRCA1/2-targeting siRNAs. Cell survival was measured using CellTiter-Glo. Data are mean ± SEM normalized to *ALC1*^*+/+*^ cells (n = 3 independent biological experiments). (C–E) The monofunctional glycosylase MPG drives MMS sensitivity in ALC1-deficient cells. (C) The monofunctional glycosylase MPG catalyzes the removal of alkylated bases, creating an abasic (AP) site. (D) Immunoblot of WCEs in *ALC1*^*+/+*^ and *ALC1*^*−/−*^ eHAP transfected with non-targeting or MPG-targeting siRNAs, probed for ALC1 and MPG. α-tubulin was used as a loading control. (E) MMS survival of *ALC1*^*+/+*^*ALC1*^*−/−*^ eHAP cells transfected with non-targeting or MPG-targeting siRNAs. Data are mean ± SEM normalized to untreated cells (n = 3 independent biological experiments). (F–H) Processing of endogenous lesions by MPG contributes to synthetic lethality with BRCA1/2 in ALC1-deficient cells. (F) Immunoblot of WCEs in *ALC1*^*+/+*^ and *ALC1*^*−/−*^ eHAP transfected with non-targeting, MPG, BRCA1/2-targeting siRNAs, probed with ALC1, BRCA1, BRCA2, and MPG. α-tubulin was used as a loading control. (G) Representative images (n = 3 biologically independent experiments) of clonogenic survival assays in *ALC1*^*+/+*^ and *ALC1*^*−/−*^ eHAP cells transfected with non-targeting, MPG, and BRCA1/2-targeting siRNAs. (H) Quantification of clonogenic survival assays in *ALC1*^*+/+*^and *ALC1*^*−/−*^ eHAP cells transfected with non-targeting, MPG, and BRCA1/2-targeting siRNAs. Data are mean ± SEM normalized to non-treated *ALC1*^*+/+*^ (n = 3 biologically independent experiments).

We next tested if SMUG1 processing of endogenous lesions underpins the synthetic lethality of cells deficient for both ALC1 and BRCA1/2. Notably, SMUG1 knockout did not significantly rescue synthetic lethality in *ALC1*^*−/−*^ cells subject to depletion of BRCA1/2 (Figures 7A and 7B). These data exclude uracil misincorporation as the predominant endogenous lesion responsible for synthetic lethality observed in ALC1- and HR-deficient cells.

Prompted by these findings, we asked if MPG, a monofunctional glycosylase responsible for the excision of alkylated base damage (Figure 7C), is responsible for MMS sensitivity in *ALC1*^*−/−*^ cells. Strikingly, depletion of MPG resulted in significant suppression of MMS sensitivity in *ALC1*^*−/−*^ cells (Figures 7D and 7E) and conferred moderate rescue of formyl-dU and Olaparib sensitivity (Figures S7A and S7B). Knockdown of MPG also resulted in a significant rescue of synthetic lethality in ALC1- and BRCA1/2-deficient cells (Figures 7F–7H) and suppressed Olaparib sensitivity in BRCA1/2-depleted cells (Figures S7C–S7H). These data reveal that processing of endogenous alkylated base damage by the glycosylase MPG creates a toxic lesion that contributes to both synthetic lethality with ALC1 deficiency and PARPi sensitivity in HR-deficient cells.

## Discussion

Alternative therapeutic strategies are needed to exploit DNA repair vulnerabilities in cancers and to mitigate innate and acquired resistance to existing treatments (Noordermeer and van Attikum, 2019). This study defines ALC1 as a compelling therapeutic target, as its loss confers PARPi sensitization, synthetic lethality with HRD, and a synergistic interaction with ATM deficiency while being largely dispensable for organismal viability.

Our findings that knockout of PARP1 and PARP2 rescued PARPi sensitivity and did not confer synthetic lethality in *ALC1*^*−/−*^ cells (Figures 2E–2K) show that PARPi sensitivity in ALC1-deficient cells is caused by increased PARP trapping, but not loss of PARP enzymatic activity per se. We also attribute PARPi sensitization to loss of nucleosome sliding activity of ALC1 (Figure 2L) but exclude that this is due to a loss of BER activity, as loss of BER is additive with ALC1 deficiency (Figures S3A–S3G). *ALC1*^*−/−*^ cells are also synthetic lethal with HRD (Figures 4C, 4D, and 4F; Figure S4O), which is associated with elevated levels of genome instability and the accumulation of ssDNA gaps at replication forks (Figures 5A–5E). Promoting or restoring HR through the removal of 53BP1 or PARG is sufficient to rescue PARPi sensitization of *ALC1*^*−/−*^ cells and synthetic lethality with *BRCA1* (Figures 2I and 4G). Notably, loss of ATM, which is a frequent event in cancers (Choi et al., 2016), confers synthetic growth defects and PARPi sensitization in the absence of ALC1 (Figures 4I–4L). Our survival analysis in breast cancer patients suggests that tumors with high ALC1 expression combined with low levels of BRCA2 have a poor prognosis (Figures 4M and 4N). Together, our data suggest that removal or inhibition of ALC1 could be exploited in HRD- and/or ATM-deficient cancers, either alone or in combination with PARPi.

Loss of ALC1 also confers sensitivity to formyl-dU and synthetic growth defects with loss of DUT (Figures 3D and 6B), the enzyme that limits the intracellular pools of dUTP and thereby minimizes uracil misincorporation during DNA replication. Contrary to our expectation, sensitivity to formyl-dU but not to PARPi or MMS in *ALC1*^*−/−*^ cells can be rescued by removing SMUG1 (Figure 6E; Figures S6F and S6G). This implies that SMUG1 is responsible for creating toxic lesions in response to formyl-dU specifically in *ALC1*^*−/−*^ cells. Loss of HR proteins BRCA2, UBC13, or ATM further sensitize *ALC1*^*−/−*^ cells to formyl-dU (Figures 6F–6H), indicating that—similar to MMS—these lesions place a critical dependence on HR in the absence of ALC1.

Since ALC1 recruitment occurs downstream of SMUG1 but upstream of APEX1 in response to formyl-dU, our data suggest that PARP1/2 and ALC1 recognize the AP site created by SMUG1. Rather than remodeling the nucleosome to allow SMUG1 access to the lesion, our data suggest that nucleosome remodeling by ALC1 is required for the effective handover from SMUG1 to APEX1. Disruption of this process through loss of either ALC1 or APEX1 leads to the accumulation of toxic lesions. While the exact nature of these lesions remains unclear, our data suggest that toxicity does not arise simply from the generation of AP sites alone, as excision of dU by UNG did not result in toxicity in either *ALC1*^*+/+*^ or *ALC1*^*−/−*^ cells (Figures 6A). Interestingly, SMUG1 has been proposed to catalyze base incision, resulting in a 3′-α,β unsaturated aldehyde and a 5′ phosphate (Alexeeva et al., 2019). This incision intermediate is subsequently removed from the 3′ end by APEX1. It is therefore possible that the accumulation of this SMUG1-dependent incision intermediate drives formyl-dU toxicity in both ALC1- and APEX1-deficient cells. Alternatively, remodeling by ALC1 could be required for the efficient release of SMUG1 from chromatin. Indeed, SMUG1 has been shown to bind with high affinity to the AP site following excision of uracil, which has been proposed to inhibit APEX1 activity (Pettersen et al., 2007).

While the action of SMUG1 underpins the formyl-dU sensitivity of *ALC1*^*−/−*^cells, removal of endogenous uracil lesions by SMUG1 does not explain the synthetic lethality we observed between ALC1 and HR. This suggests that a relatively low number of endogenous uracil lesions are processed by SMUG1 and implicates an alternative lesion as the source of synthetic lethality with HR. By analogy, we reasoned that sensitivity to MMS could be driven via a similar mechanism through the action of the glycosylase MPG, which excises alkylation damage. Indeed, knockdown of MPG was sufficient to rescue MMS sensitivity in ALC1^*−/−*^ cells (Figures 7C–7E; Figures S7C and S7D). This led us to ask whether the endogenous alkylation damage could be responsible for synthetic lethality observed in cells with both ALC1 and HR deficiencies. Indeed, knockdown of MPG suppressed the synthetic lethality between ALC1 and HR deficiencies and rescued PARPi sensitivity in HR-deficient cells. Hence, we propose that processing of endogenous alkylated base lesions by MPG creates toxic lesions that underpin both synthetic lethality between ALC1 and HR and PARPi sensitivity in HR-deficient cells.

In conclusion, this study shows that ALC1-dependent nucleosome remodeling is required for the efficient handover between DNA glycosylases, PARP1/2, and APEX1 downstream of lesion excision. While ALC1 loss is compatible with viability and fertility and is not pro-tumorigenic at an organismal level (Figure 1), loss of either ALC1 or APEX1 results in sensitivity to MMS, formyl-dU, and PARPi and synthetic lethality with HRD or ATM loss. Our data suggest that this is driven by the accumulation of toxic BER intermediates resulting from lesion excision by a specific glycosylase. Importantly, we identify processing of alkylated base damage by MPG as a key driver of synthetic lethality with HRD in *ALC1*^*−/−*^ as well as PARPi sensitivity in HRD. Taken together with PARPi hyper-sensitization and minimal predicted toxicity of removing or inhibiting ALC1, our work raises the possibility that selective small-molecule ALC1 inhibitors or degraders could provide an important therapeutic option in HRD- or ATM-deficient cancers, either alone or as a means to enhance PARPi sensitivity.

## STAR★methods

### Key Resources Table

**Table:** 

REAGENT or RESOURCE	SOURCE	IDENTIFIER
**Antibodies**		

RPA-pSer33	Bethyl	Cat#A300-246A, RRID:AB_2180847
RPA	Abcam	Cat#Ab2175, RRID:AB_302873
gH2AX	Millipore	Cat#05-636, RRID:AB_309864
ATM-pSer1981	Millipore	Cat#05-740, RRID:AB_309954
ATM	Sigma Aldrich	Cat#A1106, RRID:AB_796190
Chk1	Cell Signaling Technology	Cat#2360, RRID:AB_2080320
Chk1-pSer345	Cell Signaling Technology	Cat#133D3, RRID:AB_331212
Chk2	Millipore	Cat#05-649, RRID:AB_2244941
Cleaved Caspase 3	Cell Signaling Technology	Cat#9661, RRID:AB_2341188
BRCA1	Millipore	Cat#OP107, RRID:AB_213254
BRCA2	Millipore	Cat#OP95, AB_206776
ALC1/CHD1L	Cell Signaling Technology	Cat#13460, RRID:AB_2798225
MBD4	Invitrogen	Cat#PA5-51670, RRID:AB_2643787
SMUG1	Abcam	Cat#ab192240
dUTPase/DUT	Abcam	Cat#ab137097
α-Tubulin	Sigma-Aldrich	Cat#T6199, RRID:AB_477583
UNG	Novus Biologicals	Cat#NBP1-49985, RRID:AB_10012175
PARP1	Cell Signaling Technology	Cat#9542, RRID:AB_216073
UBC13/UBE2N	Cell Signaling Technology	Cat#4919, RRID:AB_2211168
PARP2	Sigma-Aldrich	Cat#MABE18, RRID:AB_10807040
Histone H3	Abcam	Cat#ab10799, RRID:AB_470239
53BP1	Bethyl Laboratories	Cat#A300-272A, RRID:AB_185520
PAR binding reagent	Millipore	Cat#MBE1031
ALC1/CHD1L (mouse)	St John’s laboratory	Cat#STJ116477
53BP1	Novus Biologicals	Cat#NB100-304, RRID:AB_10003037
RAD51	Millipore	Cat#ABE257, RRID:AB_10850319
Beta-Actin (AC-15)	Sigma-Aldrich	Cat#A1978, RRID:AB_476692
BARD1	Abcam	Cat#ab64164, RRID:AB_1924804
SMC1 antibody	Abcam	Cat# ab21583, RRID:AB_2192477
DAPI	Life Technology	Cat#D21490
Goat anti-Mouse Immunoglobulins/HRP	Agilent-Dako	Cat#P0447, RRID:AB_2617137
Swine anti-Rabbit Immunoglobulins/HRP	Agilent-Dako	Cat#P0399, RRID:AB_2617141

**Bacterial and Virus Strains**		

*E. coli* Rosetta (DE3) Competent Cells	Novagen(Merck)	Cat#0954-3CN
One Shot *ccd*B Survival 2 T1^R^ Competent Cells	ThermoFisher	Cat#A10460
One Shot Stbl3 Chemically Competent *E. coli*	ThermoFisher	Cat#C737303

**Critical Commercial Assays**		

CellTiter-Glo	Promega	Cat#G8462

**Chemicals, Peptides, and Recombinant Proteins**		

Doxycycline	Sigma-Aldrich	Cat#M0503-5X2MG
Blasticidin	ThemoFisher Scientific	Cat#A1113903
Hygromycin B	ThemoFisher Scientific	Cat#10687010
Zeocin	ThemoFisher Scientific	Cat#R25005
Puromicin	ThemoFisher Scientific	Cat#A1113803
Lipofectamine 2000	ThemoFisher Scientific	Cat#11668019
EDTA-free Complete protease inhibitor cocktail	Roche	Cat#COEDTAF-RO
PhosSTOP phosphatase inhibitor cocktail	Roche	Cat#PHOSS-RO
4x NuPAGE LDS sample buffer	ThemoFisher Scientific	Cat#NP0008
ProLong Gold antifade with DAPI	Thermo Fisher Scientific	Cat#P36931
Lipofectamine RNAiMAX	Invitrogen	Cat#13778150
QIAquick PCR purification kit	QIAGEN	Cat#28106
QIAquick Gel Extraction Kit	QIAGEN	Cat#28706
QIAprep Spin Miniprep Kit	QIAGEN	Cat# 27106
Veliparib	Selleck Chemicals	Cat#S1004
Olaparib	Selleck Chemicals	Cat#S1060
Talazoparib	Selleck Chemicals	Cat#S7048
Etoposide	Sigma Aldrich	Cat#BP885
Cisplatin	Sigma Aldrich	Cat#C2210000
Aphidicolin	Sigma Aldrich	Cat#A0781-1MG
Hydroxyurea (HU)	Sigma Aldrich	Cat#H8627-5G
Camptothecin	Sigma Aldrich	Cat#C9911
Methyl methanesulfonate (MMS)	Sigma Aldrich	Cat#129925-5G
PARGi	Sigma Aldrich	Cat#PDD00017273
dU	Sigma Aldrich	Cat#D5412
5-FU	Sigma Aldrich	Cat#6627
Formy-dU	Gift from Stephen West	NA
INDOLE-3-ACETIC ACID (IAA)	Sigma-Aldrich	Cat#I2886
Resazurin	Sigma-Aldrich	Cat#R7017
Doxycycline hyclate	Sigma-Aldrich	Cat#D9891
Subcellular Protein Fractionation Kit	Thermo Fisher	Cat# 78840
Clarity Western ECL	Bio-Rad	Cat#1705061
Clarity Max Western ECL	Bio-Rad	Cat#1705062
Mononucleosomes	EpiCypher	Cat No. 16-0006
Recombinant human PARG protein	Lambrecht et al., 2015	N/A
Recombinant human PARP1 protein	Gibbs-Seymour et al., 2016	N/A
Recombinant human ALC1 macro domain a.a 585-897	This paper	N/A
HiLoad 16/600 Superdex 200 pg	Sigma-Aldrich	GE28-9893-35
Benzonase Nuclease	Millipore-Merck	E1014
Ni-NTA Agarose	QIAGEN	30230
QuikChange Lightning Site-Directed Mutagenesis Kit	Agilent	210519
NAD+[32P]	Perkinelmer	NEG023X500UC
IPTG	Sigma-Aldrich	I6758-5G
Lysozyme	Sigma-Aldrich	62971-10G-F
Olaparib	Enzo Life Sciences	LKT-O4402-M005
Q5 Site-Directed Mutagenesis Kit	New England BioLabs	Cat#E0554
Diethylnitrosamine	Sigma-Aldrich	N0756

**Deposited Data**		

Code	GitHub	https://github.com/saphir746/ALC1-HR-survival
Mendeley Data	Mendeley	https://doi.org/10.17632/xhw58f995c.1

**Experimental Models: Cell Lines**		

Mouse: ALC1 +/+ MEFs #18	This Paper	N/A
Mouse: ALC1 +/+ MEFs #19	This Paper	N/A
Mouse: ALC1 −/− MEFs #11	This Paper	N/A
Mouse: ALC1 −/− MEFs #14	This Paper	N/A
Human: eHAP iCAS9 #3 ALC1+/+ #1 (Non-targeting gRNA LentiGuide Hygro)	This Paper	N/A
Human: eHAP iCAS9 #3 ALC1−/− #11(ALC1 EX2 gRNA LentiGuide Hygro)	This Paper	N/A
Human: U2OS Flp-In T-Rex HOST	Durocher lab	N/A
Human: ALC1 −/− U2OS Flp-In T-REx	This Paper	N/A
Human: eHAP iCAS9 #3 ALC1+/+ #1 Non-targeting gRNA LentiGuide Puro	This Paper	N/A
Human: eHAP iCAS9 #3 ALC1−/− #11 Non-targeting gRNA LentiGuide Puro	This Paper	N/A
Human: eHAP iCAS9 #3 ALC1+/+ #1 PARP1 gRNA LentiGuide Puro	This Paper	N/A
Human: eHAP iCAS9 #3 ALC1−/− #11 PARP1 gRNA LentiGuide Puro	This Paper	N/A
Human: eHAP iCAS9 #3 ALC1+/+ #1 PARP2 gRNA LentiGuide Puro	This Paper	N/A
Human: eHAP iCAS9 #3 ALC1−/− #11 PARP2 gRNA LentiGuide Puro	This Paper	N/A
Human: eHAP iCAS9 #3 ALC1+/+ #1 53BP1 gRNA LentiGuide Puro	This Paper	N/A
Human: eHAP iCAS9 #3 ALC1−/− #11 53BP1 gRNA LentiGuide Puro	This Paper	N/A
Human: eHAP iCAS9 #3 ALC1+/+ #1 pLenti CMV Puro (control)	This Paper	N/A
Human: eHAP iCAS9 #3 ALC1−/− #11 pLenti CMV Puro (control)	This Paper	N/A
Human: eHAP iCAS9 #3 ALC1−/− #11 pLenti CMV ALC1 CRISPR-resistant Puro	This Paper	N/A
Human: eHAP iCAS9 #3 ALC1−/− #11 pLenti CMV ALC1 G750E CRISPR-resistant Puro	This Paper	N/A
Human: eHAP iCAS9 #3 ALC1−/− #11 pLenti CMV ALC1 K77R CRISPR-resistant Puro	This Paper	N/A
Human: eHAP iCAS9 #3 Non-targeting gRNA LentiGuide Hygro	This Paper	N/A
Human: eHAP iCAS9 #3 ALC1 EX2 gRNA LentiGuide Hygro	This Paper	N/A
Human: eHAP iCAS9 #3 ALC1+/+ #1 POLQ gRNA LentiGuide Puro	This Paper	N/A
Human: eHAP iCAS9 #3 ALC1−/− #11 POLQ gRNA LentiGuide Puro	This Paper	N/A
Human: eHAP iCAS9 #3 ALC1+/+ #1 POLB gRNA LentiGuide Puro	This Paper	N/A
Human: eHAP iCAS9 #3 ALC1−/− #11 POLB gRNA LentiGuide Puro	This Paper	N/A
Human: eHAP iCAS9 #3 ALC1+/+ #1 FEN1 gRNA LentiGuide Puro	This Paper	N/A
Human: eHAP iCAS9 #3 ALC1−/− #11 FEN1 gRNA LentiGuide Puro	This Paper	N/A
Human: eHAP iCAS9 #3 ALC1+/+ #1 EXO1 gRNA LentiGuide Puro	This Paper	N/A
Human: eHAP iCAS9 #3 ALC1−/− #11 EXO1 gRNA LentiGuide Puro	This Paper	N/A
Human: eHAP iCAS9 #3 ALC1+/+ #1 LIG4 gRNA LentiGuide Puro	This Paper	N/A
Human: eHAP iCAS9 #3 ALC1−/− #11 LIG4 gRNA LentiGuide Puro	This Paper	N/A
Human: eHAP iCAS9 #3 ALC1+/+ #1 LIG1 gRNA LentiGuide Puro	This Paper	N/A
Human: eHAP iCAS9 #3 ALC1−/− #11 LIG1 gRNA LentiGuide Puro	This Paper	N/A
Human: eHAP iCAS9 #3 ALC1+/+ #1 LIG3 gRNA LentiGuide Puro	This Paper	N/A
Human: eHAP iCAS9 #3 ALC1−/− #11 LIG3 gRNA LentiGuide Puro	This Paper	N/A
Human: eHAP iCAS9 #3 ALC1+/+ #1 HPF1 gRNA LentiGuide Puro	This Paper	N/A
Human: eHAP iCAS9 #3 ALC1−/− #11 HPF1 gRNA LentiGuide Puro	This Paper	N/A
Human: DLD-1 ALC1 NT LentiCRISPR Puro	This Paper	N/A
Human: DLD-1 BRCA2−/− NT LentiCRISPR Puro	This Paper	N/A
Human: DLD-1 ALC1 EX2 LentiCRISPR Puro	This Paper	N/A
Human: DLD-1 BRCA2−/− ALC1 EX2 NT LentiCRISPR Puro	This Paper	N/A
Human: eHAP iCAS9 #3 ALC1+/+ #1 UBC13 gRNA LentiGuide Puro	This Paper	N/A
Human: eHAP iCAS9 #3 ALC1−/− #11 UBC13 gRNA LentiGuide Puro	This Paper	N/A
Human: eHAP iCAS9 #3 ALC1+/+ #1 ATM gRNA LentiGuide Puro	This Paper	N/A
Human: eHAP iCAS9 #3 ALC1−/− #11 ATM gRNA LentiGuide Puro	This Paper	N/A
Human: eHAP iCAS9 #3 ALC1+/+ #1 DUT gRNA LentiGuide Puro	This Paper	N/A
Human: eHAP iCAS9 #3 ALC1−/− #11 DUT gRNA LentiGuide Puro	This Paper	N/A
Human: eHAP iCAS9 #3 ALC1+/+ #1 SMUG1 gRNA LentiGuide Puro	This Paper	N/A
Human: eHAP iCAS9 #3 ALC1−/− #11 SMUG1 gRNA LentiGuide Puro	This Paper	N/A
Human: eHAP iCAS9 #3 ALC1+/+ #1 UNG gRNA LentiGuide Puro	This Paper	N/A
Human: eHAP iCAS9 #3 ALC1−/− #11 UNG gRNA LentiGuide Puro	This Paper	N/A
Human: eHAP iCAS9 #3 ALC1+/+ #1 MBD4 gRNA LentiGuide Puro	This Paper	N/A
Human: eHAP iCAS9 #3 ALC1−/− #11 MBD4 gRNA LentiGuide Puro	This Paper	N/A
Human: eHAP iCAS9 #3 ALC1−/− #11 pLenti CMV Puro (control) #2	This Paper	N/A
Human: eHAP iCAS9 #3 ALC1−/− #11 pLenti CMV ALC1 Puro	This Paper	N/A
Human: eHAP iCAS9 #3 ALC1+/+ #1 SMUG1 −/−	This Paper	N/A
Human: eHAP iCAS9 #3 ALC1−/− #11 SMUG1 −/−	This Paper	N/A
Human: eHAP iCAS9 #3 ALC1+/+ #1 APEX1 −/−	This Paper	N/A
Human: eHAP iCAS9 #3 ALC1−/− #11 APEX1 −/−	This Paper	N/A
Human: HCT116 BARD1^AID/AID^	Nakamura et al., 2019	https://doi.org/10.1038/s41556-019-0282-9
Human: HCT116 53BP1^−/−^ BARD1^AID/AID^	Becker et al., 2020 (bioRxiv)	https://doi.org/10.1101/2020.06.01.127951

**Experimental Models: Organisms/Strains**		

Mouse: Chd1lGt(E305F08)Wrst	This Paper	MGI:3910467

**Oligonucleotides**		

ALC1 G750E F: GGGCAGAGGTGAGTTATTTACAGCTC	This Paper	NA
ALC1 G750E R: CAGTGGCCAGAGTCATCT	This Paper	NA
ALC1 EX2 CRISPR-R F:ATTAGAAGGCGGAGT AAACTGGCTCGCC	This Paper	NA
ALC1 EX2 CRISPR-R R:TGATAGCTCCTTAG GTGAATCCCTGTCAGC	This Paper	NA
BRCA1 siGENOME smart-pool	Dharmacon	M-003461-02
BRCA2 siGENOME smart-pool	Dharmacon	M-003462-01
MPG ON-TARGETplus	Dharmacon	L-005146-00-0005
Non-targeting ON-TARGETplus	Dharmacon	D-001810-10
ALC1KO-3, TTTCTGCCAGGTGGATTAGG; ALC1KO-4, ATACCCTGCTTGCCATGAAA; ALC1KO-5, ATTCTGGCAATGGAAGCACT	This paper	N/A
CRISPR Guides	This Paper	Table S1
Sequencing Barcodes	This Paper	Table S2

**Recombinant DNA**		

pLenti CMV Puro DEST (w118-1)	Addgene	#17452
ALC1 CRISPR-R CMV Puro DEST	This Paper	NA
ALC1 G750E CRISPR-R CMV Puro DEST	This Paper	NA
ALC1 K77R CRISPR-R CMV Puro DEST	This Paper	NA
ALC1wt CMV Puro DEST	This Paper	NA
BFP/GFP Cas9 reporter	Addgene	#67980
px459v2	Addgene	#62988
LentiCRISPRv2	Addgene	#52961
Lenti-sgRNA-Hygro	Addgene	#104991
Lenti-sgRNA-Puro	Addgene	#104990
pX458	Addgene	#48138
Edit-R inducible lentiviral Cas9	Horizon Discovery	#CAS11229
CRISPR Guides	This Paper	Table S1
pNIC-CTHF-ALC1 585-897	This paper	N/A
pNIC-CTHF-ALC1 585-897(D723A)	This paper	N/A

**Software and Algorithms**		

Fiji	NIH	https://imagej.net/Fiji/Downloads
Image Lab 5.2.1	Bio-Rad Laboratories	http://www.bio-rad.com/en-uk/product/image-lab- software?ID = KRE6P5E8Z
Adobe Illustrator 23.11	Adobe	https://www.adobe.com/uk/products/illustrator.html
Adobe Photoshop 20.0.08	Adobe	https://www.adobe.com/uk/products/photoshop.html
Prism 8	GraphPad Software	https://www.graphpad.com/
BWA	Li and Durbin, 2009	0.5.9-r16
MAGeck	Li et al., 2014	0.5.7
R	https://www.r-project.org	3.6.3 (2020-02-29) “Holding the Windsock”
QuPath-0.2.3	https://doi.org/10.1038/s41598-017-17204-5	https://qupath.github.io/

**Other**		

High fat diet	Teklad	TD.06414
Uncropped Data	Mendeley	https://data.mendeley.com/datasets/xhw58f995c/draft?a=f322441d-e12e-4a47-b15b-95d388b35ea1

### Resource Availability

#### Lead contact

Further information and requests for resources and reagents should be directed to and will be fulfilled by the Lead Contact, Simon Boulton: simon.boulton@crick.ac.uk

#### Materials availability

Materials associated with the paper are available upon request to Lead Contact, Simon Boulton: simon.boulton@crick.ac.uk

#### Data and code availability

The code generated during this study are available at GitHub: https://github.com/saphir746/ALC1-HR-survival. Original data for figures in the paper are available at https://doi.org/10.17632/xhw58f995c.1.

### Experimental Model and Subject Details

#### Animals

Mice deficient for ALC1 were generated using an ES cell line Chd1l^Gt(E305F08)Wrst^ available from the German Gene Trap Consortium (GGTC) in which a gene-trap vector rsFROSAbgeo0s containing a β-Geo cassette was inserted between exon 1 and 2. The precise localization of the gene-trap vector was determined by GGTC and is located at position 4827 in intron 1. Chd1l^Gt(E305F08)Wrst^ ES cells were injected into C57BL/6Jax host blastocysts and implanted into pseudopregnant females. Chimeric mice were obtained and bred to SV129 mice. The resulting heterozygous (*Alc1*^*+/−*^) mice were bred to obtain homozygous *Alc1*^*−/−*^. Genotyping of the offspring was confirmed by PCR using the following primers (ALC1KO-3, TTTCTGCCAGGTGGATTAGG; ALC1KO-4, ATACCCTGCTTGCCATGAAA; ALC1KO-5, ATTCTGGCAATGGAAGCACT). For longevity studies, mice were allowed to age and observed for development of disease. The endpoint of the study was set at 23 months but if they appeared unhealthy or got palpable tumors beforehand, animals were sacrificed. They were then subjected to full necropsy.

For epithelial liver tumor development, 2-week-old mice received a single intraperitoneal (i.p.) injection of diethylnitrosamine (DEN; Sigma-Aldrich) dissolved in saline at a dose of 25 mg/kg body weight. After weaning, mice have been fed with increasing proportion of high fat diet (Teklad, TD.06414) mixed with normal chow over a 4-week period. All mice were closely monitored and allowed to reach 36 weeks old at the time where livers were harvested and fixed in 10% NBF for further histological analysis.

All animal experimentations were undertaken in compliance with UK Home Office legislation under the Animals (Scientific Procedures) Act 1986 under project license number 70/8527 and following the ARRIVE guidelines.

For longevity studies, groups of 30 mice of each genotype (Alc1^+/+^ versus Alc1^−/−^) were allowed to age and observed for development of disease. The endpoint of the study was set at 23 months but if they appeared unhealthy or got palpable tumors beforehand, animals were sacrificed. They were then subjected to full necropsy. The sample size was defined using statistical power analysis (power of 80%–90% with a significance level of 5% (p = 0.05)). Mice culled due to nonspecific phenotypes (e.g., dermatitis, overgrown teeth and fits) were excluded from this study. Most mice were housed as group of 2-3 per cages. Females were pooled to avoid single mouse housing and a small proportion of males was single housed.

Randomization was undertaken to remove any biases. Each mouse had a specific ID number that does not indicate the mouse genotype and were handled blindly by qualified animal technicians throughout the longevity study. Necropsy was done by certified histopathologists who blindly looked at H&E sections of a selection of organs and wrote a histology report for each mouse.

For the DEN exposure, sample sizes were determined by power calculations. We were not pursuing lower penetrance phenotypes, thus statistically significant data could typically be obtained with around 6 mice per group (age matched mice of a single genotype), plotted with 95% CI and statistical significance of phenotype-specific differences determined by unpaired Student’s t test. 6 breeding pairs were set up to produce the necessary mice (males only that are Alc1^+/+^ versus Alc1^−/−^). 14 days after their birth, all male pups were injected with DEN and then genotyped at the time of weaning (3 weeks of age). Females were sacrificed. Only males with the relevant genotype (Alc1^+/+^ versus Alc1^−/−^) were kept. 2 weeks after weaning, all mice were fed with a gradual increasing amount of High Fat Diet mixed with normal chow (25%, 50%, 75%, 100%; each step lasted for 1 week). All mice were fed with 100% high fat diet until they reached the age of 36 weeks. Mice were checked by abdominal palpation at least once a week for the first 5 months and then twice a week. At the end of the experiment, liver of each mouse was harvested and fixed for 24 h in NBF10%. Nodules were counted by eye and each visible nodule measured with a calliper. Histology of each liver was then performed blindly by a qualified histopathologist who counted the number of tumors and identified their type following the INHAND nomenclature of the hepatobiliary system (Thoolen et al.,. 2010).

#### Cell lines

Mouse embryonic fibroblasts (MEFs) have been derived at 13.5dpc using standard protocol and cultured in Dulbecco’s modified Eagle’s medium (DMEM) (Invitrogen) supplemented with 15% fetal bovine serum and 1% penicillin-streptomycin (Invitrogen). MEFs immortalized by Large T-SV40 were maintained with 15% FBS. The human haploid chronic myeloid leukemia cell line, eHAP (Essletzbichler et al., 2014), was purchased from Horizon Discovery (#C669) and maintained in IMDM medium (GIBCO/Thermo Fisher) supplemented with 10% FBS and Pen/Strep. U2OS Flp-In T-REx were a kind gift from Durocher lab and maintained in DMEM medium (GIBCO/Thermo Fisher) supplemented with 10% FBS and Pen/Strep. Wild-type and *BRCA2*^*KO*^ DLD-1 cells were purchased from Horizon and maintained in DMEM medium (GIBCO/Thermo Fisher) supplemented with 10% FBS and Pen/Strep. All cell lines were grown at 37 °C and 5% CO_2._

### Method Details

#### Histology, immunohistochemistry

For histology and post-mortem tissues, samples were fixed in 10% Neutral buffered formalin (NBF), paraffin embedded, sectioned at 4 μm and stained with hematoxylin and eosin. For immunohistochemistry, samples were prepared using standard methods. In brief, tissue sections were processed for staining by microwaving in 0.01M citrate buffer, pH 6. After incubation with primary antibodies (Cleaved caspase 3, Cell Signaling, #9664; γH2AX, Millipore #AB5535), samples were incubated with biotinylated secondary antibody (Vector) followed by incubation with Avidin Biotin Complex (Vector); slides were developed in 3,3′-diaminobenzidine (DAB) substrate (Vector) and counterstained in hematoxylin. Tumors and lymphomas images were taken using a Nikon Digital Sight DS-Ri1 camera paired to a Nikon 90i Eclipse microscope. Imaging software was NIS-Elements AR Ver 4.0, 64bit.

#### Lentiviral and transduction

To produce lentivirus, 4 × 10^6^ 293FT cells in a 10-cm dish were transfected with packaging plasmids (2.83 μg pLP1, 1.33 μg pLP2 and 1.84 μg pLP/VSVG) along with 5 μg of expression plasmid using 20 μL Lipofectamine 2000 (Life Technologies/Thermo Fisher) as per the manufacturer’s instructions. Medium was refreshed 12–16 h later. Virus-containing supernatant was collected ∼36–40 h post transfection, cleared through a 0.45-μm filter, supplemented with 8 μg ml^−1^ polybrene (Sigma) and used for infection of target cells. The following antibiotics were used for selection of transductants: puromycin (eHAP 0.4 μg ml^−1^; DLD-1 2 μg ml^−1^ each for 48–72 h), hygromycin (eHAP 400 μg ml^−1^ for ∼6-10 days) and blasticidin (8 μg ml^−1^, 4–5 days for all cell lines).

#### Plasmids

G750E mutation was introduced by Q5 site directed mutagenesis (NEB) in human ALC1 pDon221 (Ahel et al., 2009) using primers: F-GGGCAGAGGTGAGTTATTTACAGCTC, R-CAGTGGCCAGAGTCATCT. CRISPR resistant silent mutations were introduced into ALC1, ALC1 K77R and ALC1 G750E pDon221 by Q5 site directed mutagenesis (NEB) using primers: F-ATTAGAAGGCGGAGTAAACTGGCTCGCC, R-TGATAGCTCCTTAGGTGAATCCCTGTCAGC. Expression vectors were made in pLenti CMV Puro DEST (w118-1) (Addgene#17452) using the Gateway system (Life Technologies/Thermo Fisher) according to the manufacturer’s protocol. Expression constructs were introduced into ALC1 ^−/−^ eHAP cells by lentiviral transduction.

#### RNA interference

BRCA1 was targeted with 50 nM siGENOME smart-pool (M-003461-02). BRCA2 was targeted with 50 nM siGENOME smart-pool (M-003462-01). MPG was targeted with 50 nM ON-TARGETplus (L-005146-00-0005). 50 nM Non-targeting ON-TARGETplus (D-001810-10) pool was used as a control. For double siRNA transfections 25 nM of each target or control siRNA was used. siRNA oligonucleotides were transfected in Opti-MEM reduced-serum medium using RNAiMAX (Life Technologies/Thermo Fisher). Following siRNA transfection, cells were seeded either for survival assays (24 h post transfection) or for immunofluorescence, cell cycle, metaphase, replication fiber and immunoblot analysis (48 h post transfection).

#### DNA damaging drugs

PARP inhibitors olaparib, talazoparib and veliparib were purchased from Selleck Chemicals. Methyl methanesulfonate (MMS), 5-Flurouracil, HU, CPT, etoposide, cisplatin and aphidicolin were obtained from Sigma. Concentrations and durations of treatment are indicated in the sections below and in the respective figures.

#### Generation of Dox-inducible Cas9-expressing cells

eHAP iCAS9 cells were transduced with the Edit-R inducible lentiviral Cas9 vector (Horizon Discovery) and transductants were selected with blasticidin. Single cell clones were then seeded by limiting dilution in a 96 well plate. Cas9 editing efficiency and Dox regulation was tested as follows. Cells were transduced at a low (∼0.3) multiplicity of infection (MOI) with BFP/GFP Cas9 reporter (Addgene #67980). Cells were split into ± 1μg/mL Dox 24 h following transduction. Dox containing media was replenished 48 h following transduction. Cells were analyzed on LRSII BD Bioscience. BFP positive sells were gated to select cells which had been transduced. The percentage of GFP positive cells was then calculated. Clones were selected that had a low % (< 5%) GFP positive in the +Dox condition to select for high Cas9 editing activity and high % (> 95%) GFP positive in the -Dox condition to select clones with tight regulation (Figure S2K).

#### Generation of CRISPR knockout cell lines

sgRNAs targeting the following sequences are listed in (Table. 1). Guides were cloned into px459v2 (Addgene #62988), px458 (Addegene #48138), LentiCRISPRv2 (Addgene #52961), Lenti-sgRNA-Hygro (Addgene #104991) or Lenti-sgRNA-Puro (Addgene #104990) as indicated (Table. 1). ALC1^+/+^ and ALC1^−/−^ eHAP were generated by transducing eHAP iCAS9 generated as described above with NT gRNA or ALC1 gRNA cloned into Lenti-sgRNA-Hygro (Table. 1). Cells were selected in Hygromycin at a concentration of 400 μg ml^−1^. The resulting NT gRNA and ALC1 gRNA iCAS9 cell lines were then banked. To make individual knockout clones, cells were treated with 1 μg/mL Dox for 72 h and then seeded as single cell clones by limiting dilution. The resulting plates were duplicated and screened using IF for ALC1. Knockout clones were then confirmed by immunoblotting against ALC1. ALC1^−/−^ U2OS Flp-In T-REx cell lines were generated by the transient transfection of cells with px459 containing guides against ALC1. Clones were isolated and screened as above. Inducible CRISPR knockout cell lines were generated by transducing ALC1^+/+^ and ALC1^−/−^ iCAS9 cells with lentivirus produced from the sgRNA constructs listed in (Table. 1) followed by antibiotic selection. Knockout of target proteins was confirmed by immunoblotting following 72 h 1 μg/mL Dox. SMUG1 and APEX1 KO cell lines were created by transiently transfecting ALC1^+/+^ and ALC1^−/−^ iCAS9 cells with APEX1 LentiGuide puro and SMUG1 LentiGuide puro. Cells were pulsed with 0.4ug/mL puro for 2 days and Cas9 expression was induced by treating with 1μg/mL Dox for 72 h. Clones were isolated and screened as above.

#### Whole-cell extracts, SDS-PAGE and immunoblotting

For whole cell lysates, PBS washed cells were lysed in RIPA Buffer (10 mM Tris-Cl pH 8.0, 1 mM EDTA, 0.5 mM EGTA, 1% Triton X-100, 0.1% sodium deoxycholate, 0.1% SDS, 140 mM NaCl, 1x phosphatase (Phos-Stop, Roche) and protease (Complete, EDTA-free, Roche) inhibitor mixes) on ice for 20 min. Lysates were sonicated with a probe at medium intensity for 5 s in a Soniprep 150 instrument and clarified by centrifugation at 13000 g for 15 min at 4°C. Protein concentration was quantified using the DC Protein Assay (Bio-Rad) according to the manufacturer’s instructions. Proteins were denatured in 2X NuPAGE LDS sample buffer (Invitrogen) and 1% 2-metcaptoethanol (Sigma-Aldrich) for 5 min at 95°C. Proteins were separated by SDS-PAGE using NuPAGE mini gels (Invitrogen) and transferred onto 0.2 μm pore Nitrocellulose membrane (Amersham Protran; Sigma-Aldrich). Membranes were blocked with 5% skim milk/TBST (TBS/0.1%Tween-20) for 1 h at room temperature and probed with the indicated primary antibodies overnight at 4°C. Membranes were then washed 3 times for 10 min with TBST, incubated with appropriate secondary antibodies conjugated to a horseradish peroxidase (HRP) for 1 h at room temperature and washed again 3 times for 10 min with TBST. Immunoblots were developed using Clarity or Clarity Max Western ECL Substrate (Bio-Rad).

#### PARP Trapping

Subcellular fractionation was performed using the Subcellular Protein Fractionation Kit for Cultured Cells (Cat# 78840, Thermo Fisher) according to the manufacturer’s instructions. Chromatin fractions corresponding to 1 × 10^6^ cells were compared to whole cell lysates corresponding to 150 000 cells, and separated by SDS-PAGE as indicated.

#### Chromatin Fractionation

4x10^6^ eHAP cells were seeded per 10cm dish 24 h prior to collection. Cells were treated with 10uM formyl-dU or DMSO control for 1 h prior to collection. Cells were scraped in 1ml ice-cold PBS. 50% of the sample was kept on ice for whole cell control. The remaining cells were spun down for 4 min at 500 g and resuspended in 200ul CSK buffer (10mM PIPES pH7.0, 100mM NaCl, 300mM Sucrose, 1.5mM MgCL_2_, 5mM EDTA, 0.5% Triton 1x phosphatase (Phos-Stop, Roche) and protease (Complete, EDTA-free, Roche) inhibitor mixes)) and incubated on ice for 10 min. Cells were spun down at full speed for 10 s 150ul of supernatant (soluble fraction) was collected. Residual soluble fraction was removed and the chromatin pellet was washed in 500ul of CSK. Whole cell chromatin pellets were resuspended in 200ul 1X NuPAGE LDS sample buffer (Invitrogen) and 1% 2-metcaptoethanol (Sigma-Aldrich). 50uL of 4x NuPAGE LDS sample buffer (Invitrogen) and 4% 2-metcaptoethanol (Sigma-Aldrich) was added to soluble fraction. Samples were sonicated with a probe at medium intensity for 10 s in a Soniprep 150 instrument and then incubated at 95°C for 10 min. 20ul of each fraction was loaded and subjected to SDS-PAGE as above.

#### CRISPR–Cas9 screening

CRISPR screens were performed as described(Doench et al., 2016). eHAP iCAS9 expressing NT or ALC1 gRNA were transduced with the lentiviral Brunello library (Addgene #73179-LV) at a low MOI (∼0.2–0.3) in 2 biologically independent transductions. Puromycin-containing medium was added the next day to select for transductants. Selection was continued until 72 h post transduction. At this point the cells from transduction #2 were split into two technical 2 replicates giving 3 replicates in total. Cells were then subcultured in 1 μg/mL Dox to induce CAS9 expression for 144 h. Following this, pellets of 40 million cells were collected from each replicate for sequencing of T1. Each of the three replicates was divided into two populations. One was left untreated and to the other 250 nM Olaparib was added. Cells were grown with or without Olaparib for a further 144 h and subcultured every two days. Sample cell pellets were frozen at each time point for genomic DNA (gDNA) isolation. A library coverage of ^3^400 cells per sgRNA was maintained at every step.

gDNA from cell pellets was isolated using the QIAamp Blood Maxi Kit (QIAGEN) and genome-integrated sgRNA sequences were amplified by ExTaq polymerase (Takara) using P5 and P7 multiplexing barcoded primers (Table. 2). The concentration and quality of the libraries following gel purification was measured using Qubit and TapeStation, pooled them at 4 nM and sequenced on the HiSeq 4000 with 75 bp reads. Data presented in Table.3.

#### CRISPR sequencing analysis

Raw data was trimmed by obtaining 20 bp after the first occurrence of “CACCG” in the read sequence. Trimmed reads were then mapped with BWA (version 0.5.9-r16)(Li and Durbin, 2009) to a database of guide sequences for the human CRISPR Brunello lentiviral pooled library downloaded from Addgene (https://www.addgene.org/pooled-library/broadgpp-human-knockout-brunello/) with the parameters “-l 20 -k 2 -n 2.” sgRNA counts were obtained after filtering the mapped reads for those that had zero mismatches, and mapped to the forward strand of the guide sequence. The MAGeck ‘test’ command (version 0.5.7)(Li et al., 2014) was used to perform the sgRNA ranking analysis between the relevant conditions with parameters “–norm-method total–remove-zero both.”

#### CellTiter-Glo survival assays

For eHAP and U2OS cell lines, 200 and 150 cells respectively per well were seeded in a 96 well plates, drug treatments were added 18 h following plating and cells were grown for a further 5 days. CellTiter-Glo assay (Promega) was performed as per manufacturer’s instruction. Luminescence was measured using Beckman Coulter Paradigm detection platform. For drug sensitivity, treated cells were normalized to untreated samples.

#### Clonogenic survival assays

For eHAP clonogenic survival assays 200 cells were seeded per well of a 24-well plate in technical triplicate. Drug treatments were added 18 h following plating and cells were grown for a further 5 days. Surviving colonies were stained using crystal violet and imaged and quantified using GelCount (Oxford Optronix). For drug sensitivity, treated cells were normalized to untreated samples. For DLD-1 BRCA2^+/+^ ALC1^+/+^ and DLD1 BRCA2^+/+^ ALC1^−/−^ clonogenic survival assays, 90 cells were seeded per well of a 24-well plate in technical triplicate. Drug treatments were added 18 h following plating and cells were grown for a further 8 days. For DLD-1 BRCA2^−/−^ ALC1^+/+^, 400 cells were seeded per well of a 24-well plate in technical triplicate. Drug treatments were added 18 h following plating and cells were grown for a further 11 days. For DLD-1 BRCA2^−/−^ ALC1-low expressing, 1200 cells were seeded per well of a 24-well plate in technical triplicate. Drug treatments were added 18 h following plating and cells were grown for a further 11 days. For drug sensitivity, treated cells were normalized to untreated samples.

#### Cell cycle analysis by FACS

For EdU/PI Flow Cytometry, cells were labeled for 30 min with 10 μM EdU, fixed in 4% PFA, permeabilized in PBS-Triton 0.3% and washed in 1% BSA before samples were processed using the Click-iT EdU Flow Cytometry Cell Proliferation Assay (Thermo Fisher) with Alexa Fluor 488. DNA was counterstained with Propidium Iodide (10 μg/mL). Newly synthesized DNA (EdU) and DNA content (PI) were detected using an LSRII (Becton Dickinson). Gating of single cells and cell cycle analysis was performed manually using FlowJo (TreeStar).

#### Protein expression and purification

ALC1 macro domain proteins were expressed in *E. coli* Rosetta (DE3) cells in Lysogeny Broth supplemented with 50 μg/mL kanamycin and 34 μg/mL chloroamphenicol. Cells were induced at OD_600_ 0.6 with 0.35 mM IPTG and grown overnight at 18 °C. Cell pellets were resuspended in lysis buffer (50 mM HEPES-NaOH, pH 7.5, 500 mM NaCl, 5% glycerol, 4 mM 2-mercaptoethanol, 10mM imidazole, protease inhibitor cocktail (Roche), 25 units/mL of benzonase (Sigma-Aldrich), and 2 mg/mL lysozyme and lysed thoroughly by Emulsi Flex-C5 homogenizer (Avestin) at 15,000Psi. Lysate was centrifuged for 60 min at 35,000 g and applied to Ni-NTA agarose resin (50% slurry, QIAGEN) equilibrated to lysis buffer. The proteins were eluted by 500 mM imidazole and then puriðed over a Superdex S-200 (16/600) column in 40 mM HEPES-NaOH, pH 7.5, 200 mM NaCl and 1 mM DTT.

PARP1, PARG and HPF1 were purified as described previously(Lambrecht et al., 2015; Langelier et al., 2011; Suskiewicz et al., 2020).

#### Chromosome spreading

To facilitate the analysis of structural chromosome aberrations, cells were incubated for 5 h in medium containing 330 nM nocodazole (Sigma). Mitotic cells were swelled in a hypotonic solution (DMEM: deionized water at 1:3 ratio) for 6 min at RT. Subsequently, cells were fixed with Carnoy’s buffer (freshly made) for 15 min at RT and spun down, this fixation step was repeated four times. The suspension of cells in Carnoy’s buffer (100 μl) was dropped on a clean slide and let dry at RT. Slides were incubated with 3% Giemsa in PBS for 6 min at RT. After drying, slides were mounted with DPX mountant (Sigma). Images were acquired using an Olympus FV1000D (InvertedMicroscopeIX81) confocal laser scanning microscope equipped with a PlanApoN × 60/1.40 NA Oil Sc objective lens controlled by FV10-ASW software.

#### Immunofluorescence microscopy

eHAP cells were incubated with 1 μM EdU for 30 min before fixation. Cells were treated with pre-extraction buffer (10 mM Pipes, pH 7.0, 100 mM NaCl, 300 mM sucrose, 1.5 mM MgCl_2_, 5 mM EDTA, 0.3 mM RNase A and 0.5% Triton X-100) for 3 min on ice, then fixed with 4% formaldehyde at room temperature (RT) for 15 min. Fixed cells were stained for EdU incorporation using Click-iT EdU Imaging Kit (Life Technology C10340), then processed for immunofluorescence microscopy (IF). Primary antibodies used are listed in (Table. 3). Alexa fluorophore-conjugated secondary antibodies were used for detection. DNA was stained using DAPI. Images were acquired using a Nikon Ti2 microscope fitted with a CSU-W1 spinning disk confocal unit (Yokogawa) and a Prime 95B camera (Photometrics) using Plan Apochromat 100x/1.45 NA Oil objective lens and controlled by Nikon NIS-Elements. For RAD51, images were acquired using an Olympus FV1000D (InvertedMicroscopeIX81) confocal laser scanning microscope equipped with a PlanApoN × 60/1.40 NA Oil Sc objective lens controlled by FV10-ASW software.

#### Quantification of DNA damage markers

Quantification of DNA damage marker foci and signal intensity was performed by Fiji software.

#### DNA Fiber assay

DNA fiber assay was performed as described in(Bellelli et al., 2018). Briefly, eHAP ALC1^+/+^ and ALC1^−/−^ transfected with control siRNA or siRNA targeting BRCA1 or BRCA2, were pulse labeled with 20 μM CldU for 20 min and subsequently with 200 μM IdU for 20 min. After tripsinization and counting, cells were resuspended at a concentration of 5x 10^5^ in PBS and 2.5 μL of cell suspension were spotted on glass slides and lysed with 7.5 μL of a buffer containing 0.5% SDS, 200 mM Tris-HCL, pH 7.4, and 50 mM EDTA. Slides were then tilted to allow a stream of DNA to move slowly toward the botton of the slide, briefly air-dried and then fixed in methanol/acetic acid (3:1) (15 min at R.T). Slides were subsequently denatured in HCl 2,5 M (30 min R.T.), extensively washed in dH2O and PBS, blocked in 1% BSA/PBS (30 min R.T.) and incubated with rat anti-BrdU monoclonal antibody (1:1000 overnight; AbD Serotec) and subsequently with mouse anti-BrdU monoclonal antibody (1:500 1 h R.T.; Becton Dickinson). After incubation with a mixture of Alexa Fluor 488 rabbit anti-mouse and Alexa Fluor 594 goat anti-rat antibodies (1:500 45 min R.T.; Invitrogen) slides were mounted in PBS/Glycerol 1:1 and finally examined using Axio Imager.M2 (ZEISS) with 63x oil immersion objective and the Volocity 6.3 software.

#### Detection of ssDNA gaps by S1 nuclease DNA fiber assay

To identify the presence of ssDNA gaps on ongoing replication forks, we adapted a DNA fiber assay(Bellelli et al., 2018) to include S1 nuclease to degrade ssDNA(Quinet et al., 2017). Briefly, cells were incubated with 20 μM CldU for 30 min and subsequently pulse-labeled with 200 μM IdU for 60 min. Cells were then permeabilized with CSK-triton (0.5%) for 10 min at R.T. and then washed in PBS and S1 nuclease buffer (30 mM sodium acetate, 10 mM zinc acetate, 5% glycerol, 50 mM NaCl, pH 4.6). Samples were incubated with S1 buffer containing (or not) 20 U/mL of S1 nuclease (Invitrogen Cat #18001016) for 30 min at 37°C. After a wash in PBS/BSA 0.1%, cells were scraped, centrifuged and resuspended at a concentration of 1-2x10^3^ cells/μl. 2 μl of resuspended nuclei were pipetted on the top of a microscope slide and lysed with 8μl of lysis buffer (200 mM Tris–HCl, pH 7.5, 50 mM EDTA, 0.5% SDS). After a few minutes, slides were tilted to allow the lysate to slowly travel toward the bottom of the slide and air-dried. Slides were then fixed with Methanol-Acetic acid (3:1) for 15 min at R.T., washed with dH2O and denatured with 2.5 M HCl for 45 min at R.T. After several washes in dH2O and PBS, slides were blocked with PBS/BSA 1% for 30 min at R.T. and incubated with rat-anti BrdU antibody (Abcam, Cat# ab6326) 1/1000 overnight at 4°C and subsequently with mouse anti-BrdU (BD Biosciences, Cat# 347580) 1/500 for 1 h at R.T. After incubation with a mixture of goat anti-rat Alexa Fluor 594 (Invitrogen, Cat# A-11007) and rabbit anti-mouse Alexa Fluor 488 (Invitrogen, Cat# A-11059) slides were washed, mounted in PBS/Glycerol and images acquired using an AxioImager M1 microscope with 63x objective. Data were reported as IdU/CldU tract ratios.

#### TCGA survival analysis

##### 1 Data Acquisition

Primary and processed data, notably RNA expression levels for genes CHD1L, BRCA1, BRCA2 and ATM for breast cancer patients were downloaded from The Cancer Genome Atlas (TCGA)[ref here] in May 2020. Samples with any of the following were excluded from analysis: (1) “Not available” gene expression values, (2) insufficient survival information and (3) missing date / year of birth. As the TCGA cohort has already received Ethics Committee Approval, this study did not require additional approval. Our primary outcomes was cancer mortality at the latest follow-up available, as recorded in the TGCA dataset. Our primary exposure was RNA expression of gene CHD1L, also known as ACL1 (Chromodomain-helicase-DNA-binding protein 1-like, Chrm. 1 bp 147,242,654- 147,295,765 bps) as well as age. Secondary exposure of interest were RNA levels of the genes BRCA1, BRCA2 and ATM. No sex adjustment was performed, as all patients in our dataset were female. These processes were performed using R software version 3.5.0

##### 2 Survival Analysis

Survival analyses were performed using: (1) multivariate Cox proportional hazards regres-sion, and (2) Kaplan-Meier survival curves. These were performed using the “survival” and “survminer” packages in R. For the Cox regression, we used age as the time metric, and we regressed survival against terciles of RNA expression of ACL1 to account for potential nonlin-ear associations. Survival data was left and right censored, with left censoring set at age at diagnosis, and right censoring set at age of death or age at last follow-up. We investigated the interactive effects of ACL1 expression terciles and expression terciles of BRCA1, BRCA2 and ATM on survival, and tested for the significance of the interactive terms using likelihood ratio test. Results are quoted in Hazard Ratios (HR) per year of life. All likelihood ratio tests for interaction are performed using a set of two nested models, where the reduced model is an additive version of the interaction models. For Kaplan-Meier analyses, age was not included, neither as a confounder nor as a timescale. Instead, we used days between diagnosis and censoring as a time-scale. Survival profiles were compiled for all combinations of ACL1 gene expression terciles and BRAC1 / BRCA2 / ATM expression terciles. We present a p value for significance of survival difference between the gene expression profile. Associations between survival and variables of interest were considered significant if the p values associated with the results passed below the significance threshold p < 0.05.

### Quantification and Statistical Analysis

Sample number (n) indicates the number of independent biological samples in each experiment and are indicated in figure legends or methods. GraphPad Prism and R were used for all statistical analysis: Kaplan–Meier plots for survival and calculate significance using Log-rank (Mantel–Cox) test, unpaired t test or ANOVA multiple comparison tests were used unless stated otherwise.

## Supplemental Information

Document S1. Figures S1–S7

Table S1. CRISPR constructs and cloning oligos used to generate cell lines, related to STAR Methods

Table S2. Barcode primers used for CRISPR screen sequencing library generation, related to STAR Methods

Table S3. Gene summary and raw gRNA reads, related to Figure 3

Table S4. Tumor spectrum table, related to Figure 1

Document S2. Article plus Supplemental Information
