# Supplementary material for: Defective ALC1 nucleosome remodeling confers PARPi sensitization and synthetic lethality with HRD
Source: Mol Cell. 2021 Feb 18;81(4):767–783.e11. doi: 10.1016/j.molcel.2020.12.006 (PMC7895907; doi:10.1016/j.molcel.2020.12.006)
Supplement: Document S1. Figures S1–S7 [file mmc1.pdf]

Molecular Cell, Volume *81*

Supplemental Information

Defective ALC1 nucleosome remodeling confers PARPi sensitization and synthetic lethality with HRD

Graeme Hewitt, Valerie Borel, Sandra Segura-Bayona, Tohru Takaki, Phil Ruis, Roberto Bellelli, Laura C. Lehmann, Lucia Sommerova, Aleksandra Vancevska, Antonia Tomas-Loba, Kang Zhu, Christopher Cooper, Kasper Fugger, Harshil Patel, Robert Goldstone, Deborah Schneider-Luftman, Ellie Herbert, Gordon Stamp, Rachel Brough, Stephen Pettitt, Christopher J. Lord, Stephen C. West, Ivan Ahel, Dragana Ahel, J. Ross Chapman, Sebastian Deindl, and Simon J. Boulton

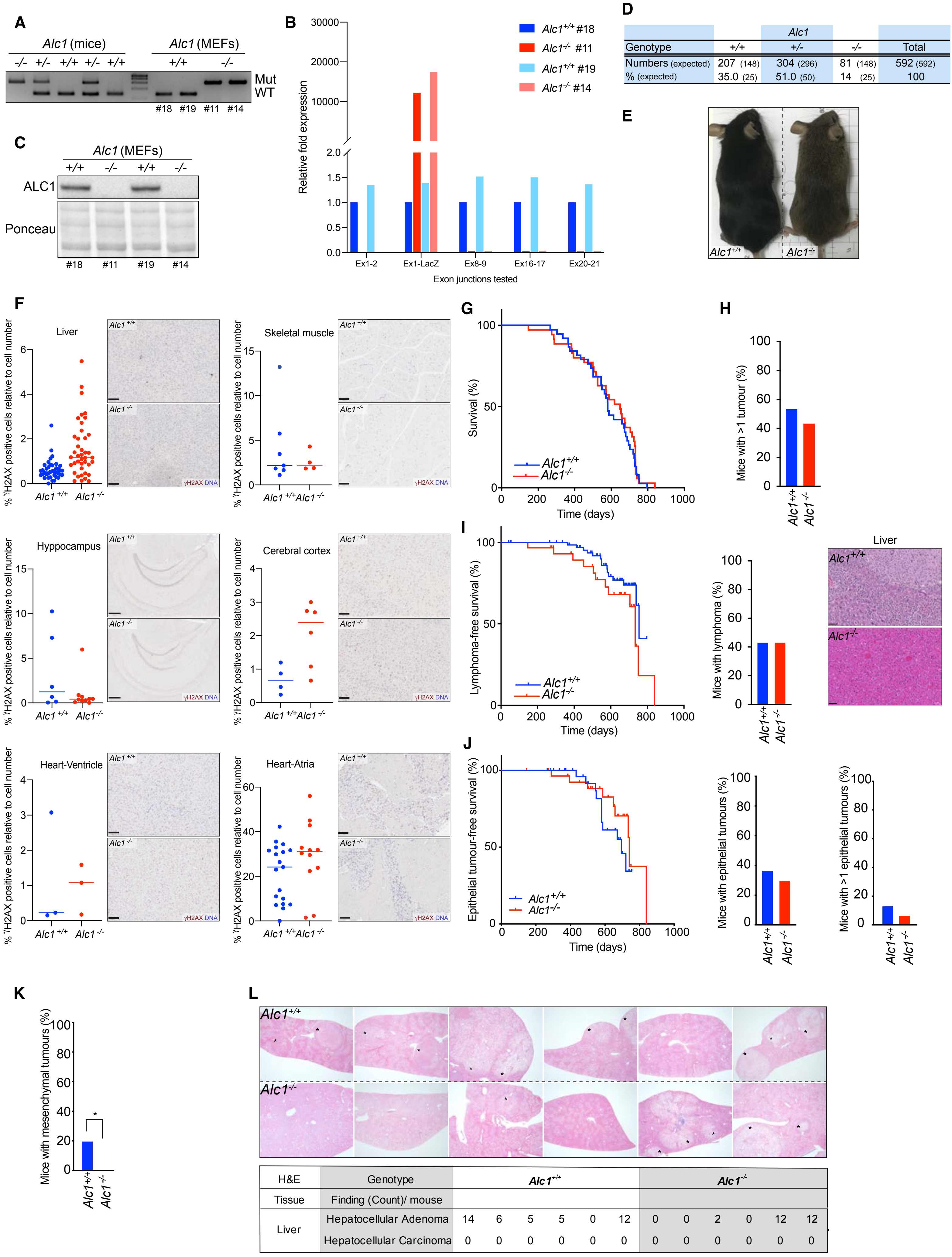

		Alc1		
Genotype	+/+	+/-	-/-	Total
Numbers (expected)	207 (148)	304 (296)	81 (148)	592 (592)
% (expected)	35.0 (25)	51.0 (50)	14 (25)	100

H&E	Genotype	*Alc1*$^{+/+}$						*Alc1*$^{-/-}$					
Tissue	Finding (Count)/ mouse												
Liver	Hepatocellular Adenoma	14	6	5	5	0	12	0	0	2	0	12	12
	Hepatocellular Carcinoma	0	0	0	0	0	0	0	0	0	0	0	0

Supplementary Figure. 1

Figure S1, relating to Figure 1. **Tumour prevalence in *Alc1$^{-/-}$* mice.**

(A) *Alc1* mice PCR genotyping strategy. Upper band represents the mutant allele; lower band, the WT allele. **(B)** Fold reduction of *Alc1* transcript in *Alc1* mice relative to *Alc1$^{+/+}$* analyzed by qRT-PCR using primers spanning the junction of different exons along the *Alc1* gene. **(C)** ALC1 protein expression in MEFs. Ponceau was used as loading control. **(D)** Mendelian ratios of *Alc1* heterozygous mice breeding. Numbers and percent in bracket are expected numbers and ratios. χ^2=54.068, p<0.0001. **(E)** Representative images of 14-week old *Alc1$^{+/+}$* and *Alc1$^{-/-}$* littermates showing a slight weight difference. **(F)** DNA damage analysis in different mitotic and post-mitotic *Alc1$^{+/+}$* and *Alc1$^{-/-}$* mice tissues. **(G)** Overall survival of *Alc1* mice. Significance: Mantel-Cox test, p= 0.6. n= 30 *Alc1$^{+/+}$* and n= 30 *Alc1$^{-/-}$*. Mice culled due to nonspecific phenotypes (e.g., dermatitis, overgrown teeth, and fits) were excluded from this study. **(H)** Frequency of *Alc1* mice which develop more than 1 tumour. Note that there is no difference between both groups. **(I)** (Left) Lymphoma-free survival of *Alc1* mice. Significance: Mantel-Cox test, p= 0.1. n= 30 *Alc1$^{+/+}$* and n= 30 *Alc1$^{-/-}$*. Mice culled due to nonspecific phenotypes (e.g., dermatitis, overgrown teeth, and fits) were excluded from this study. (Middle) Frequency of *Alc1* mice which develop lymphomas. (Right) Representative images of liver lymphoma section stained with hematoxylin and eosin. Note the presence of lymphoma in *Alc1$^{+/+}$* liver. Scale bars represent 50µm. **(J)** (Left) Epithelial tumour-free survival of *Alc1* mice. Significance: Mantel-Cox test, p= 0.2. n= 30 *Alc1$^{+/+}$* and n= 30 *Alc1$^{-/-}$*. Mice culled due to nonspecific phenotypes (e.g., dermatitis, overgrown teeth, and fits) were excluded from this study. Note the longer survival of mice lacking ALC1. (Middle) Frequency of *Alc1* mice which develop epithelial tumours. (Right) Frequency of *Alc1* mice which develop more than 1 epithelial tumour. Fisher's exact test, p= 0.6. Note the tendency for the *Alc1$^{-/-}$* mice to develop less epithelial tumours. **(K)** Frequency of *Alc1* mice which develop mesenchymal tumours. Fisher's exact test, p= 0.02. *Alc1$^{-/-}$* mice develop less mesenchymal tumours. **(L)** (Top) Representative images of haematoxylin and eosin stained liver sections following DEN-induced tumorigenesis. Asterisks identify hepatocellular adenomas. (Bottom) Quantification of the number of hepatocellular adenomas identified in each section by a board-certified veterinary pathologist. Note the tendency of the *Alc1$^{-/-}$* mice to develop less hepatocellular adenomas.

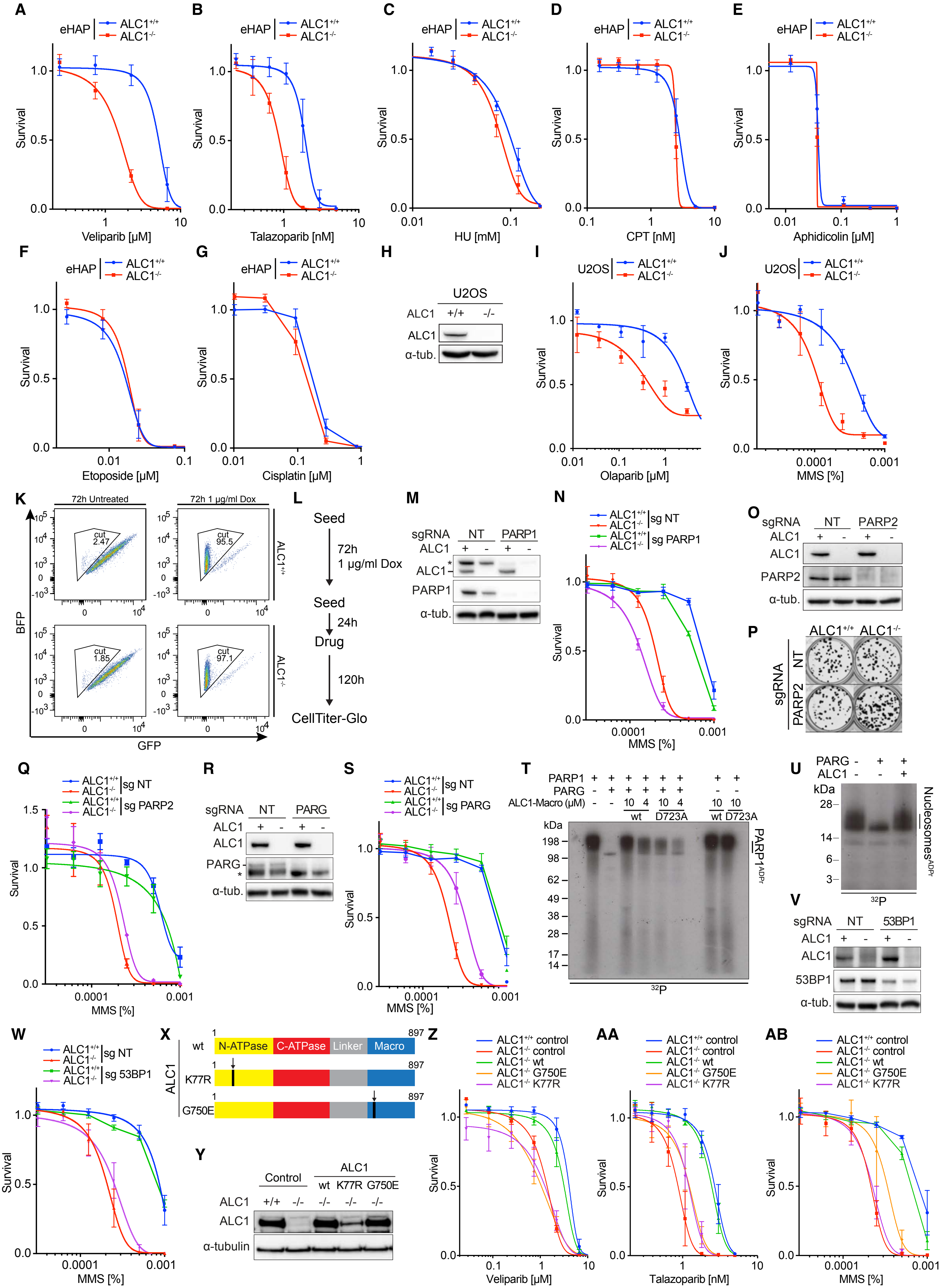

Supplementary Figure. 2

Figure S2, relating to Figure 2. Response to genotoxins and mechanisms of PARPi resistance in *ALC1*$^{-/-}$ cells.
(A,B) Reduced survival of eHAP *ALC1*$^{-/-}$ cells after treatment with indicated PARPi. Data are mean ± s.e.m normalised to untreated cells (n = 3 independent biological experiments). Solid lines show a nonlinear least-squares fit to a four-parameter dose–response model. **(C-G)** Survival of eHAP *ALC1*$^{-/-}$ cells after treatment with indicated genotoxin. Data are mean ± s.e.m normalised to untreated cells (n = 3 independent biological experiments). Solid lines show a nonlinear least-squares fit to a four-parameter dose–response model. **(H)** CRISPR-mediated inactivation of ALC1 in U2OS. Immunoblot of WCEs in *ALC1*$^{+/+}$ and *ALC1*$^{-/-}$ cells, probed for ALC1. α-tubulin was used as a loading control. **(I)** Reduced survival of U2OS ALC1$^{-/-}$ cells after treatment with Olaparib. Data are mean ± s.e.m normalised to untreated cells (n = 3 independent biological experiments). Solid lines show a nonlinear least-squares fit to a four-parameter dose–response model. **(J)** Reduced survival of U2OS ALC1$^{-/-}$ cells after treatment with MMS. Data are mean ± s.e.m normalised to untreated cells (n = 3 independent biological experiments). Solid lines show a nonlinear least-squares fit to a four-parameter dose–response model. **(K)** Single cell clones of ALC1$^{+/+}$ and ALC1$^{-/-}$ were isolated and screened for CAS9 activity using GFP-BFP reporter ±72h Dox. BFP+ cells were gated and the loss of GFP expression was measured using FACS and displayed as pseudo colour FACS plots. Gates labelled cut represent the percentage of BFP+ GFP- cells. **(L)** Schematic representation of survival assays using CellTiter-Glo in inducible CAS9 cells. **(M)** Immunoblot of WCEs from *ALC1*$^{+/+}$ and *ALC1*$^{-/-}$ iCAS9 cells expressing sgRNA against NT or PARP1 following 72h Dox, probed for ALC1 and PARP1. α-tubulin was used as a loading control. **(N)** Increased MMS sensitivity in iCAS9 ALC1$^{+/+}$ and ALC1$^{-/-}$ eHAP expressing PARP1 sgRNA following 72h Dox. Data are mean ± s.e.m normalised to untreated cells (n = 3 independent biological experiments). Solid lines show a nonlinear least-squares fit to a four-parameter dose–response model. **(O)** Immunoblot of WCEs from *ALC1*$^{+/+}$ *and ALC1*$^{-/-}$ iCAS9 cells expressing sgRNA against NT or PARP2 following 72h Dox, probed for ALC1 and PARP2. α-tubulin was used as a loading control. **(P)** Representative images (n = 3 biologically independent experiments) of clonogenic survival assays in *ALC1*$^{+/+}$ and *ALC1*$^{-/-}$ *iCAS9* cells expressing indicated sgRNA following 72h Dox. **(Q)** MMS sensitivity in iCAS9 ALC1$^{+/+}$ and ALC1$^{-/-}$ eHAP expressing PARP2 sgRNA following 72h Dox. Data are mean ± s.e.m normalised to untreated cells (n = 3 independent biological experiments). Solid lines show a nonlinear least-squares fit to a four-parameter dose–response model. **(R)** Immunoblot of WCEs from *ALC1*$^{+/+}$ *and ALC1*$^{-/-}$ iCAS9 cells expressing sgRNA against NT or PARG following 72h Dox, probed for ALC1 and PARG. α-tubulin was used as a loading control. **(S)** Rescue of MMS sensitivity in *iCAS9 ALC1*$^{-/-}$ eHAP expressing PARG sgRNA following 72h Dox. Data are mean ± s.e.m normalised to untreated cells (n = 3 independent biological experiments). Solid lines show a nonlinear least-squares fit to a four-parameter dose–response model. **(T)** Radioactive ADP-ribosylation assay of PARP1 ± PARG incubated with macro-ALC1(wt or mutant) at the indicated concentrations. **(U)** Radioactive ADP-ribosylation assay of nucleosomes ± PARG incubated with macro-ALC1. **(V)** Immunoblot of WCEs from *ALC1*$^{+/+}$ and *ALC1*$^{-/-}$ *iCAS9* cells expressing sgRNA against NT or 53BP1 following 72h Dox, probed for ALC1 and 53BP1. α-tubulin was used as a loading control. **(W)** MMS sensitivity in inducible CAS9 *ALC1*$^{+/+}$ and *ALC1*$^{-/-}$ eHAP expressing NT or 53BP1 sgRNA following 72h Dox. Data are mean ± s.e.m normalised to untreated cells (n = 3 independent biological experiments). **(X-AB)** PARPi and MMS sensitivity are associated with defective nucleosome remodelling. **(X)** Schematic of domain structure of indicated ALC1 constructs. **(Y)** Immunoblot of WCEs in *ALC1*$^{+/+}$ and *ALC1*$^{-/-}$ cells transduced with indicated constructs, probed for ALC1. α-tubulin was used as a loading control. **(Z,AA)** PARPi sensitivity in *ALC1*$^{+/+}$ and *ALC1*$^{-/-}$ cells transduced with the indicated constructs. Data are mean ± s.e.m normalised to untreated cells (n = 3 independent biological experiments). Solid lines

show a nonlinear least-squares fit to a four-parameter dose–response model. **(AB)** MMS sensitivity in *ALC1*$^{+/+}$ and *ALC1*$^{-/-}$ cells transduced with the indicated constructs. Data are mean ± s.e.m normalised to untreated cells (n = 3 independent biological experiments). Solid lines show a nonlinear least-squares fit to a four-parameter dose–response model.

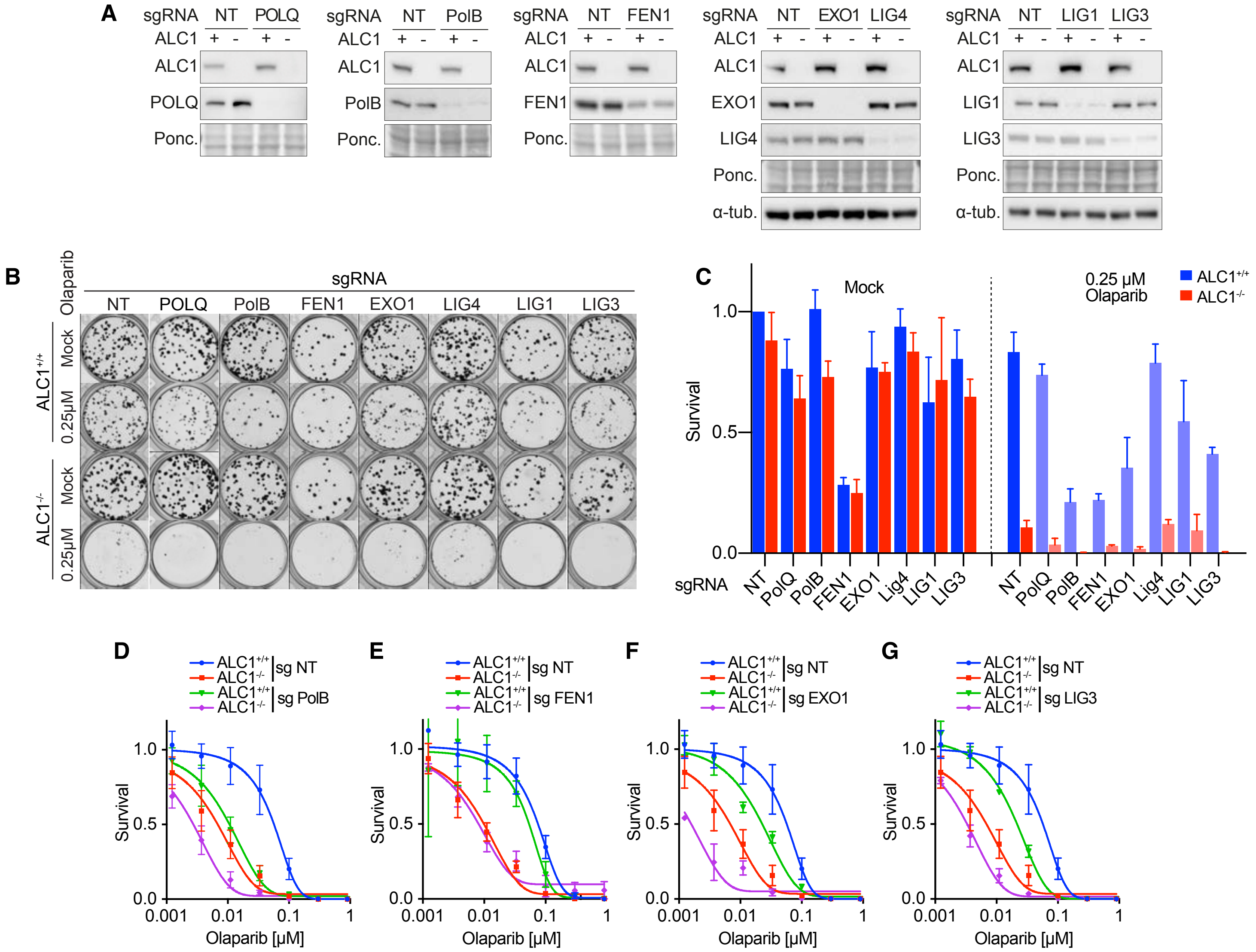

Supplementary Figure. 3

Figure S3, relating to Figure 3. **DDR candidate *ALC1$^{-/-}$* cell panel.**
(A) Immunoblots of WCEs in *ALC1$^{+/+}$* and *ALC1$^{-/-}$ iCAS9* cells transduced with indicated sgRNA following 72h Dox. Probed with indicated antibodies. **(B)** Representative images (n = 3 biologically independent experiments) of clonogenic survival assays in *ALC1$^{+/+}$* and *ALC1$^{-/-}$ iCAS9* cells expressing indicated sgRNA following 72h Dox ± 250 nM Olaparib. **(C)** Quantification of clonogenic survival assays in *ALC1$^{+/+}$ and ALC1$^{-/-}$ iCAS9* cells expressing indicated sgRNA following 72h Dox ± 250 nM Olaparib. Data are mean ± s.e.m normalised to non-treated *ALC1$^{+/+}$* NT sgRNA (n = 3 biologically independent experiments). **(D)** Olaparib survival of *ALC1$^{+/+}$* and *ALC1$^{-/-}$ iCAS9* cells transduced with NT sgRNA and Polβ sgRNA following 72h Dox. Data are mean ± s.e.m normalised to untreated cells (n = 3 independent biological experiments). Solid lines show a nonlinear least-squares fit to a four-parameter dose–response model. **(E)** Olaparib survival of ALC1$^{+/+}$ and ALC1$^{-/-}$ iCAS9 cells transduced with NT sgRNA and FEN1 sgRNA following 72h Dox. Data are mean ± s.e.m normalised to untreated cells (n = 3 independent biological experiments). Solid lines show a nonlinear least-squares fit to a four-parameter dose–response model. **(F)** Olaparib survival of *ALC1$^{+/+}$* and *ALC1$^{-/-}$ iCAS9* cells transduced with NT sgRNA and EXO1 sgRNA following 72h Dox. Data are mean ± s.e.m normalised to untreated cells (n = 3 independent biological experiments). Solid lines show a nonlinear least-squares fit to a four-parameter dose–response model. **(G)** Olaparib survival of *ALC1$^{+/+}$* and *ALC1$^{-/-}$ iCAS9* cells transduced with NT sgRNA and LIG3 sgRNA following 72h Dox. Data are mean ± s.e.m normalised to untreated cells (n = 3 independent biological experiments). Solid lines show a nonlinear least-squares fit to a four-parameter dose–response model.

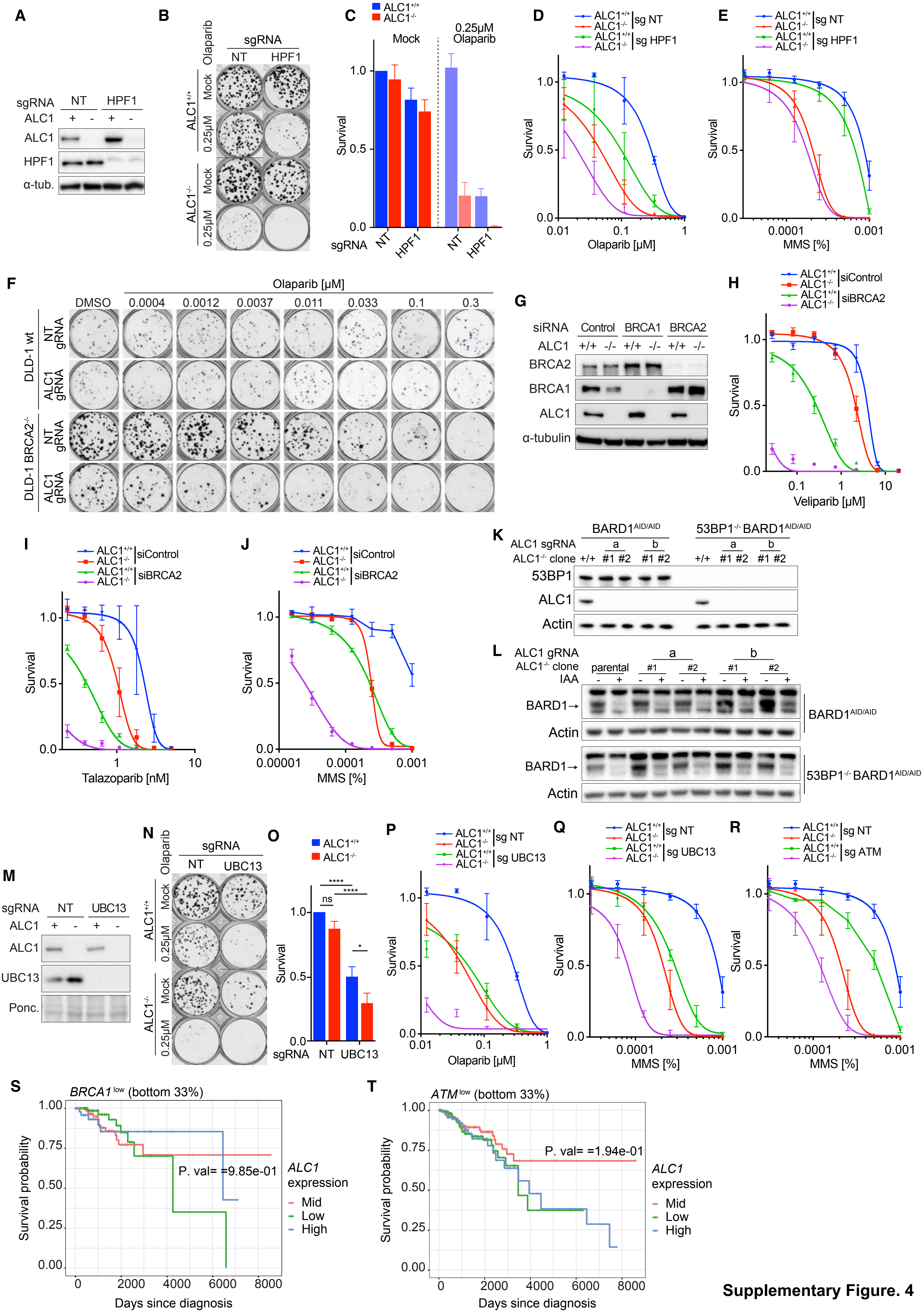

Supplementary Figure. 4

Figure S4, relating to Figure 4. PARPI hypersensitivity in ALC1-/- cells in HRD and ATM deficient cells.

(A) Immunoblots of WCEs in *ALC1*$^{+/+}$ and *ALC1*$^{-/-}$ *iCAS9* cells transduced with NT or HPF1 sgRNA following 72h Dox, probed with antibodies against ALC1 and HPF1. α-tubulin is used as a loading control. **(B)** Representative images (n = 3 biologically independent experiments) of clonogenic survival assays in *ALC1*$^{+/+}$ and *ALC1*$^{-/-}$ *iCAS9* cells expressing NT and HPF1 sgRNA following 72h Dox ± 250 nM Olaparib. **(C)** Quantification of clonogenic survival assays in *ALC1*$^{+/+}$ and *ALC1*$^{-/-}$ *iCAS9* cells expressing NT sgRNA and HPF1 sgRNA following 72h Dox ± 250 nM Olaparib. Data are mean ± s.e.m normalised to non-treated *ALC1*$^{+/+}$ NT sgRNA (n = 3 biologically independent experiments). **(D,E)** Olaparib and MMS survival of *ALC1*$^{+/+}$ and *ALC1*$^{-/-}$ *iCAS9* cells transduced with NT sgRNA and HPF1 sgRNA following 72h Dox. Data are mean ± s.e.m normalised to untreated cells (n = 3 independent biological experiments). Solid lines show a nonlinear least-squares fit to a four-parameter dose–response model. **(F)** Representative images (n = 3 biologically independent experiments) of clonogenic survival assays in DLD-1 *BRCA2*$^{+/+}$ *ALC1*$^{+/+}$*, BRCA2*$^{+/+}$ *ALC1*$^{-/-}$*, BRCA2*$^{-/-}$ *ALC1*$^{+/+}$ and *BRCA2*$^{-/-}$ *ALC1*$^{Low\ expression}$ (Fig. 4A,B) with the indicated dose of Olaparib. **(G)** Immunoblot of WCEs in *ALC1*$^{+/+}$ and *ALC1*$^{-/-}$ eHAP cells transfected with non-targeting or BRCA1/2-targeting siRNAs probed for ALC1, BRCA1 and BRCA2. α-tubulin was used as a loading control. **(H-J)** *ALC1*$^{+/+}$ and *ALC1*$^{-/-}$ eHAP cells were transfected with non-targeting or BRCA2-targeting siRNAs and treated with genotoxin indicated. Data are mean ± s.e.m normalised to untreated cells (n = 3 biologically independent experiments). Solid lines show a nonlinear least-squares fit to a four-parameter dose–response model. **(K)** Immunoblots of WCEs in parental (*ALC1*$^{+/+}$) and *ALC1*$^{-/-}$ *BARD1*$^{AID/AID}$ and parental (*ALC1*$^{+/+}$) and *ALC1*$^{-/-}$ *53BP1*$^{-/-}$*BARD1*$^{AID/AID}$ cells, probed with antibodies against ALC1 and 53BP1. Actin is used as a loading control. **(L)** Immunoblots of WCEs in parental and *ALC1*$^{-/-}$ in *BARD1*$^{AID/AID}$ cells (top) and parental and *ALC1*$^{-/-}$ *53BP1*$^{-/-}$*BARD1*$^{AID/AID}$ cells (bottom) ± 2h IAA, probed with antibodies against BARD1. Actin is used as a loading control. **(M-O)** Loss of UBC13 is synthetic lethal in ALC1$^{-/-}$ cells. **(M)** Immunoblot of WCEs from *ALC1*$^{+/+}$ and *ALC1*$^{-/-}$ *iCAS9* cells expressing sgRNA against NT or UBC13 following 72h Dox, probed for ALC1 and UBC13. Ponceau was used as a loading control. **(N)** Representative images (n = 3 biologically independent experiments) of clonogenic survival assays in *ALC1*$^{+/+}$ and *ALC1*$^{-/-}$ *iCAS9* cells expressing sgRNA against NT or UBC13 following 72h Dox ± 250 nM Olaparib. **(O)** Quantification of non-treated control clonogenic survival assays in *ALC1*$^{+/+}$ *and ALC1*$^{-/-}$ *iCAS9* cells expressing NT or UBC13 sgRNA following 72h Dox. Data are mean ± s.e.m normalised to *ALC1* +/+ NT sgRNA (n = 3 biologically independent experiments). **(P,Q)** Survival of *ALC1*$^{+/+}$ and *ALC1*$^{-/-}$ eHAP cells expressing NT or UBC13 sgRNA following 72h Dox treated with genotoxin indicated. Data are mean ± s.e.m normalised to untreated cells (n = 3 biologically independent experiments). Solid lines show a nonlinear least-squares fit to a four-parameter dose–response model. **(R)** MMS survival of *ALC1*$^{+/+}$ and *ALC1*$^{-/-}$ *iCAS9* cells transduced with NT sgRNA and ATM sgRNA following 72h Dox. Data are mean ± s.e.m normalised to untreated cells (n = 3 independent biological experiments). Solid lines show a nonlinear least-squares fit to a four-parameter dose–response model. **(S)** KM survival analysis of BRCA1low breast cancer patients from TGCA according to ALC1 expression.

(T) KM survival analysis of ATMlow breast cancer patients from TGCA according to ALC1 expression.

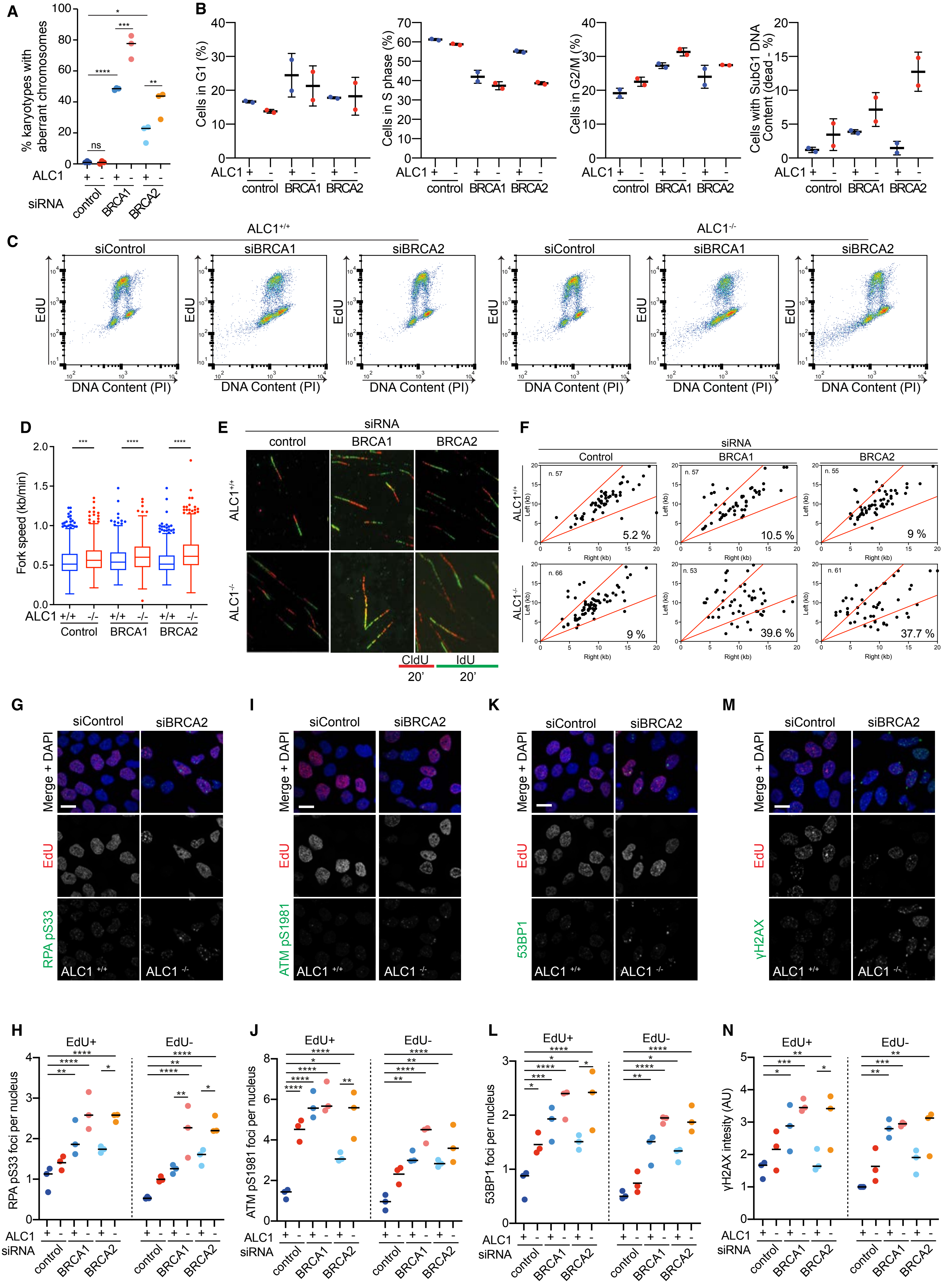

Supplementary Figure. 5

Figure S5, relating to Figure 5. Phenotypic characterisation of cell cycle, replication and DDR following loss of HR in ALC1 -/- cells.

(A) Loss of ALC1 exacerbates genomic instability in HR deficient cells. Quantification of % of karyotypes with aberrant metaphase spreads from *ALC1*$^{+/+}$ and *ALC1*$^{-/-}$ eHAP cells transfected with non-targeting, BRCA1-targeting and BRCA2-targeting siRNAs. Data are means from individual experiments; bar represents median (n = 3 independent biological experiments). **(B)** Quantification of % of cells in different cell-cycle stages in *ALC1*$^{+/+}$ and *ALC1*$^{-/-}$ eHAP cells transfected with non-targeting, BRCA1-targeting or BRCA2-targeting siRNAs. Data are n=2 independent biological experiments. **(C)** Pseudo colour FACS plots showing EdU incorporation and DNA content in *ALC1*$^{+/+}$ and *ALC1*$^{-/-}$ eHAP cells transfected with non-targeting, BRCA1-targeting or BRCA2-targeting siRNAs. Data are representative of n=2 independent biological experiments. **(D)** Boxplot showing replication fork speed (measured as IdU track length/min) in *ALC1*$^{+/+}$ and *ALC1*$^{-/-}$ eHAP transfected with the indicated siRNAs. Data are from >500 fibres, mean ± s.d from n = 2 independent biological experiments. **(E)** Lower**:** scheme of the nucleotide labelling strategy used in the fiber assay experiments. Upper: Representative DNA fiber immunofluorescence images from *ALC1*$^{+/+}$ and *ALC1*$^{-/-}$ eHAP cells transfected with the indicated siRNAs. **(F)** Analysis of replication fork symmetry from ALC1+/+ and ALC1-/- eHAP cells transfected with control siRNA or siRNAs targeting BRCA1 or BRCA2. The total number of newly activated replication forks analysed is indicated. **(G)** Representative micrographs of *ALC1*$^{+/+}$ and *ALC1*$^{-/-}$ eHAP cells transfected with non-targeting and BRCA2-targeting siRNAs stained with RPA pSer33 antibody, EdU click-iT and DAPI. Scale bar = 10 um. **(H)** Quantification of nuclear RPA pS33 foci in CSK pre-extracted EdU+ and EdU- *ALC1*$^{+/+}$ and *ALC1*$^{-/-}$ eHAP cells transfected with indicated siRNAs 72h following knockdown. Data are means from individual experiments, bar represents median (n = 3 biologically independent experiments). **(I)** Representative micrographs of *ALC1*$^{+/+}$ and *ALC1*$^{-/-}$ eHAP cells transfected with non-targeting and BRCA2-targeting siRNAs stained with ATM pS1981 antibody, EdU click-iT and DAPI. Scale bar = 10 um. **(J)** Quantification of nuclear ATM pS1981 foci in CSK pre-extracted EdU+ and EdU- *ALC1*$^{+/+}$ and *ALC1*$^{-/-}$ eHAP cells transfected with indicated siRNAs 72h following knockdown. Data are means from individual experiments, bar represents median (n = 3 biologically independent experiments). **(K)** Representative micrographs of *ALC1*$^{+/+}$ and *ALC1*$^{-/-}$ eHAP cells transfected with non-targeting, BRCA1-targeting and BRCA2-targeting siRNAs stained with 53BP1 antibody, EdU click-iT and DAPI. Scale bar = 10 um. **(L)** Quantification of nuclear 53BP1 foci in CSK pre-extracted EdU+ and EdU- *ALC1*$^{+/+}$ and *ALC1*$^{-/-}$ eHAP cells transfected with indicated siRNAs 72h following knockdown. Data are means from individual experiments, bar represents median (n = 3 biologically independent experiments). **(M)** Representative micrographs of *ALC1*$^{+/+}$ and *ALC1*$^{-/-}$ eHAP cells transfected with non-targeting and BRCA2-targeting siRNAs stained with γH2AX antibody, EdU click-iT and DAPI. Scale bar = 10 um. **(N)** Quantification of nuclear γH2AX intensity in CSK pre-extracted EdU+ and EdU- *ALC1*$^{+/+}$ and *ALC1*$^{-/-}$ eHAP cells transfected with indicated siRNAs 72h following knockdown. Data are means from individual experiments normalised to ALC1$^{+/+}$ siControl (EdU-), bar represents median (n = 3 biologically independent experiments).

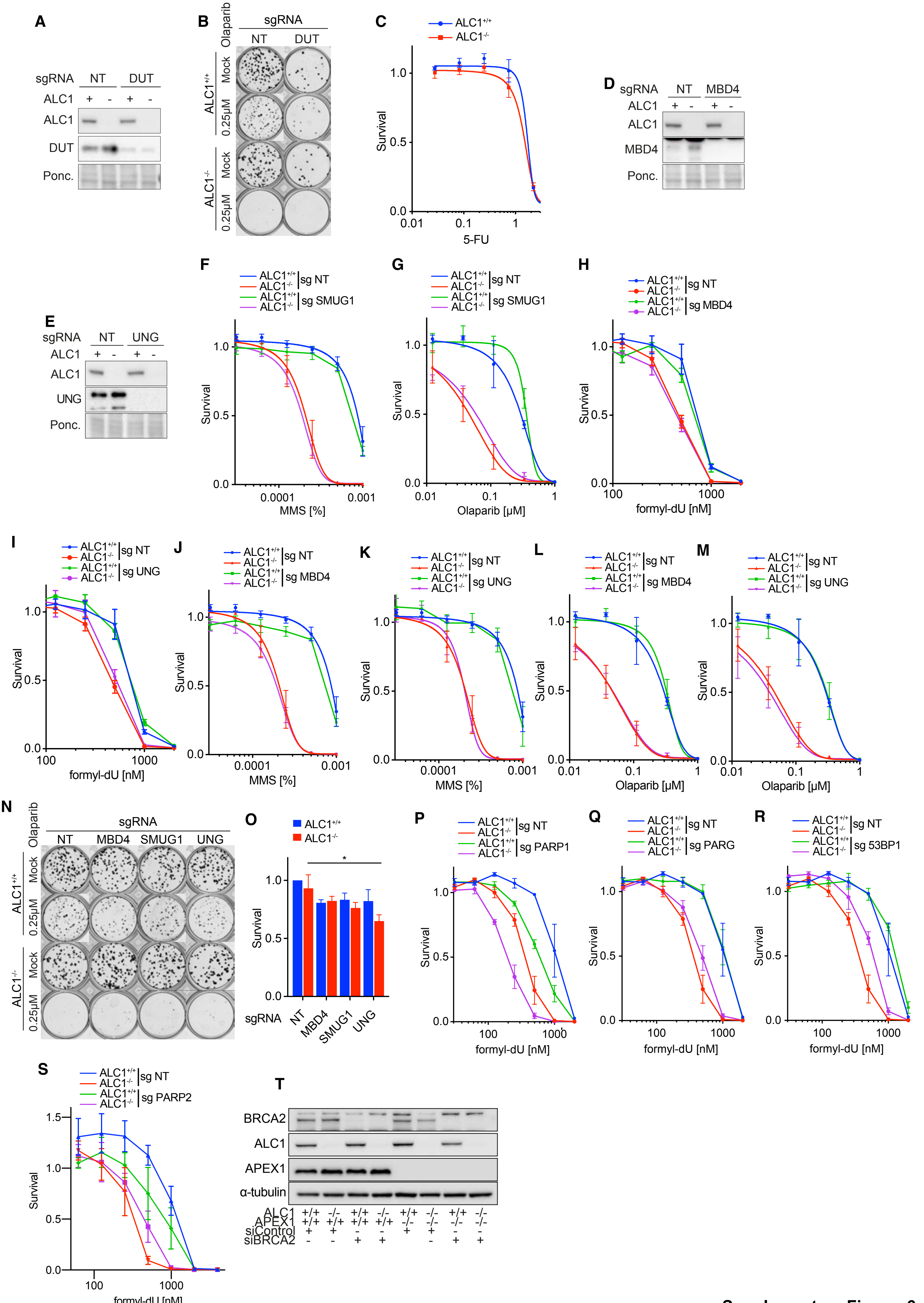

Supplementary Figure. 6

Figure S6, relating to Figure 6. Uracil metabolism in *ALC1$^{-/-}$* cells.
(A) Immunoblots of WCEs in *ALC1$^{+/+}$* and *ALC1$^{-/-}$ iCAS9* cells transduced with DUT sgRNA following 72h Dox. Probed with antibodies against ALC1 and DUT. Ponceau is used as a loading control. **(B)** Representative images (n = 3 biologically independent experiments) of clonogenic survival assays in *ALC1$^{+/+}$* and *ALC1$^{-/-}$ iCAS9* cells expressing NT and DUT sgRNA following 72h Dox ± 250 nM Olaparib. **(C)** 5-FU sensitivity in *ALC1$^{+/+}$* and *ALC1$^{-/-}$* eHAP. Data are mean ± s.e.m normalised to untreated cells (n = 3 biologically independent experiments). Solid lines show a nonlinear least-squares fit to a four-parameter dose–response model. **(D,E)** Immunoblot of WCEs *ALC1$^{+/+}$* and *ALC1$^{-/-}$* eHAP iCAS9 cells expressing indicated sgRNA following 72h Dox. Probed for ALC1, MBD4 and UNG. Ponceau was used as a loading control. **(F,G)** SMUG1 knockout does not rescue ALC1 dependent sensitivity to Olaparib or MMS. **(F)** MMS survival of *ALC1$^{+/+}$* and *ALC1$^{-/-}$ iCAS9* cells transduced with NT sgRNA and SMUG1 sgRNA following 72h Dox. Data are mean ± s.e.m normalised to untreated cells (n = 3 independent biological experiments). **(G)** Olaparib survival of *ALC1$^{+/+}$* and *ALC1$^{-/-}$ iCAS9* cells transduced with NT sgRNA and SMUG1 sgRNA following 72h Dox. Data are mean ± s.e.m normalised to untreated cells (n = 3 independent biological experiments). **(H,I)** Formyl-dU survival of *ALC1$^{+/+}$* and *ALC1$^{-/-}$ iCAS9* cells transduced with indicated sgRNA following 72h Dox. Data are mean ± s.e.m normalised to untreated cells (n = 3 independent biological experiments). **(J,K)** MMS survival of *ALC1$^{+/+}$* and *ALC1$^{-/-}$ iCAS9* cells transduced with indicated sgRNA following 72h Dox. Data are mean ± s.d normalised to untreated cells (n = 3 independent biological experiments). **(L,M)** Olaparib survival of *ALC1$^{+/+}$* and *ALC1$^{-/-}$* iCAS9 cells transduced with indicated sgRNA following 72h Dox. Data are mean ± s.d normalised to untreated cells (n = 3 independent biological experiments). **(N)** Representative images (n = 3 biologically independent experiments) of clonogenic survival assays in *ALC1$^{+/+}$* and *ALC1$^{-/-}$ iCAS9* cells expressing indicated sgRNA following 72h Dox ± 250 nM Olaparib. **(O)** Quantification of non-treated control clonogenic survival assays in *ALC1$^{+/+}$* and *ALC1$^{-/-}$ iCAS9* cells expressing indicated sgRNA following 72h Dox. Data are mean ± s.e.m normalised to *ALC1$^{+/+}$* NT sgRNA (n = 3-5 biologically independent experiments). **(P)** Increased formyl-dU sensitivity in *iCAS9 ALC1$^{+/+}$* and *ALC1$^{-/-}$* eHAP expressing PARP1 sgRNA following 72h Dox. Data are mean ± s.e.m normalised to untreated cells (n = 3 independent biological experiments). **(Q)** Formyl-dU sensitivity in *iCAS9 ALC1$^{-/-}$* eHAP expressing PARG sgRNA following 72h Dox. Data are mean ± s.e.m normalised to untreated cells (n = 3 biologically independent experiments). **(R)** Partial rescue of formyl-dU sensitivity in *iCAS9 ALC1$^{-/-}$* eHAP expressing 53BP1 sgRNA following 72h Dox. Data are mean ± s.e.m normalised to untreated cells (n = 3 biologically independent experiments). **(S)** Partial rescue of formyl-dU sensitivity in *iCAS9 ALC1$^{-/-}$* eHAP expressing PARP2 sgRNA following 72h Dox. Data are mean ± s.e.m normalised to untreated cells (n = 3 biologically independent experiments). **(T)** Immunoblot of WCEs in *ALC1$^{+/+}$ APEX1 $^{+/+}$*, *ALC1$^{+/+}$ APEX1 $^{-/-}$*, *ALC1$^{-/-}$ APEX1 $^{+/+}$*and *ALC1$^{-/-}$ APEX1 $^{-/-}$* eHAP cells transfected with non-targeting or BRCA2-targeting siRNAs, probed for ALC1, APEX1 and BRCA2. α-tubulin was used as a loading control.

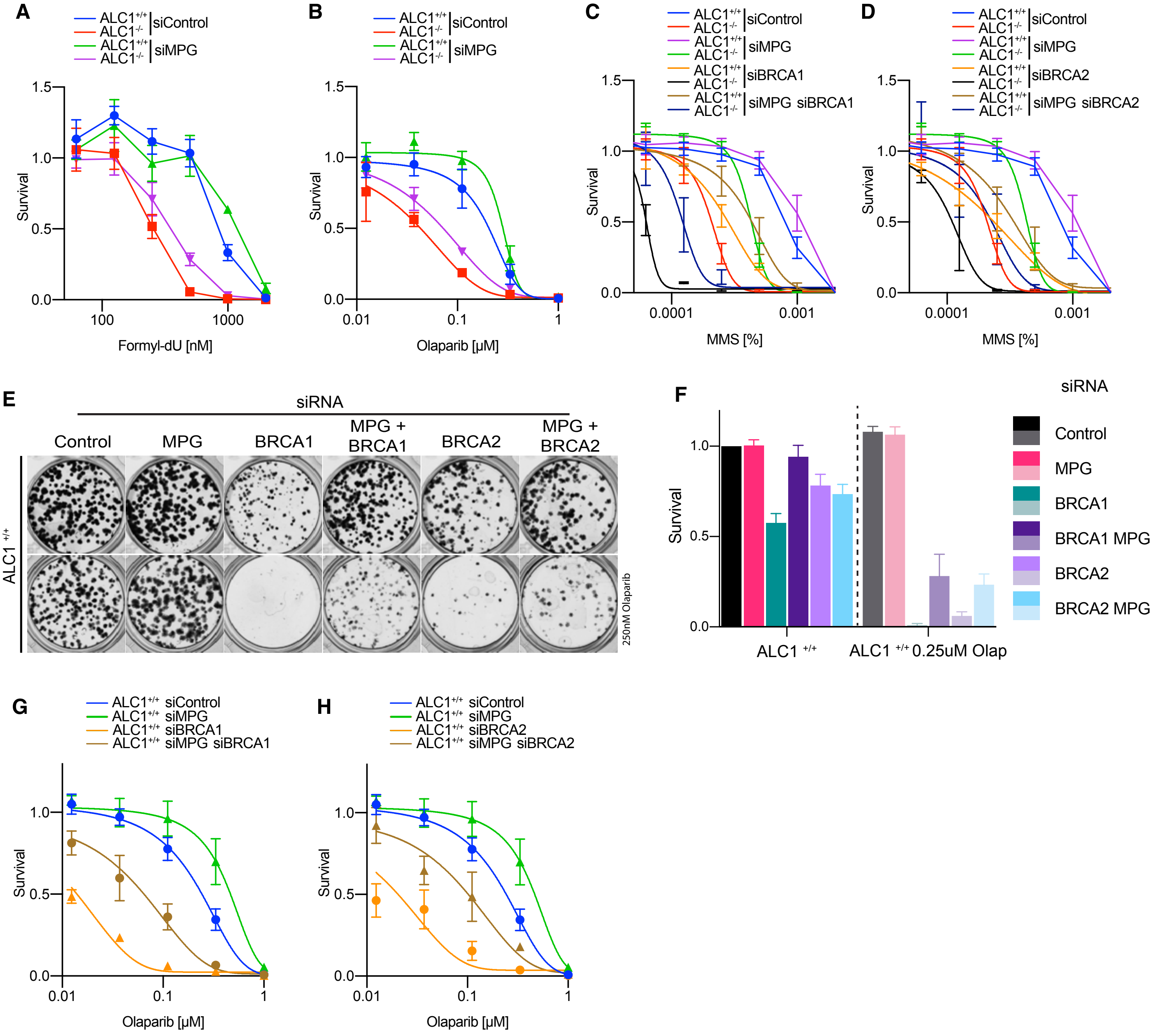

Supplementary Figure. 7

Figure S7, relating to Figure 7. The role of MPG in MMS and PARPi response in ALC1$^{-/-}$ cells.
(A) Formyl-dU survival of *ALC1*$^{+/+}$ and *ALC1*$^{-/-}$ eHAP cells transfected with non-targeting or MPG-targeting siRNAs. Data are mean ± s.e.m normalised to untreated cells (n = 3 independent biological experiments). **(B)** Olaparib survival of *ALC1*$^{+/+}$ and *ALC1*$^{-/-}$ eHAP cells transfected with non-targeting or MPG-targeting siRNAs. Data are mean ± s.e.m normalised to untreated cells (n = 3 independent biological experiments). Solid lines show a nonlinear least-squares fit to a four-parameter dose–response model. **(C)** MMS survival of *ALC1*$^{+/+}$ and *ALC1*$^{-/-}$ eHAP cells transfected with non-targeting, BRAC1 and MPG-targeting siRNAs. Data are mean ± s.e.m normalised to untreated cells (n = 3 independent biological experiments). Solid lines show a nonlinear least-squares fit to a four-parameter dose–response model. **(D)** MMS survival of *ALC1*$^{+/+}$ and *ALC1*$^{-/-}$ eHAP cells transfected with non-targeting, BRAC2 and MPG-targeting siRNAs. Data are mean ± s.e.m normalised to untreated cells (n = 3 independent biological experiments). Solid lines show a nonlinear least-squares fit to a four-parameter dose–response model. **(E)** Representative images (n = 3 biologically independent experiments) of clonogenic survival assays in *ALC1*$^{+/+}$ eHAP cells transfected with non-targeting, MPG and BRCA1/2-targeting siRNAs ± 250 nM Olaparib. **(F)** Quantification of clonogenic survival assays in ALC1$^{+/+}$ eHAP cells transfected with non-targeting, MPG and BRCA1/2-targeting siRNAs ± 250 nM Olaparib. Data are mean ± s.e.m normalised to non-treated *ALC1*$^{+/+}$ non-targeting siRNA (n = 3 biologically independent experiments). **(G)** Olaparib survival of *ALC1*$^{+/+}$ eHAP cells transfected with non-targeting, BRCA1 and MPG-targeting siRNAs. Data are mean ± s.e.m normalised to untreated cells (n = 3 independent biological experiments). Solid lines show a nonlinear least-squares fit to a four-parameter dose–response model. **(H)** Olaparib survival of *ALC1*$^{+/+}$ eHAP cells transfected with non-targeting, BRCA2 and MPG-targeting siRNAs. Data are mean ± s.e.m normalised to untreated cells (n = 3 independent biological experiments). Solid lines show a nonlinear least-squares fit to a four-parameter dose–response model.
